# Considering Ecosystem Services in Food System Resilience

**DOI:** 10.3390/ijerph19063652

**Published:** 2022-03-19

**Authors:** Yevheniia Varyvoda, Douglas Taren

**Affiliations:** 1 Mel and Enid Zuckerman College of Public Health, The University of Arizona, Tucson, AZ 85724, USA; 2 School of Medicine, University of Colorado, Aurora, CO 80045, USA; douglas.taren@cuanschutz.edu

**Keywords:** ecosystem services, resilience, natural hazards, food system, smallholder farming

## Abstract

The prevalence and severity of natural hazards pose a serious risk to food systems, undermining their function to provide food security and improved nutrition. The impact of such events is extensive, and the level of damage and recovery significantly depend on ecosystem services, including their own resilience capacity. This paper provides evidence that the role, value, and utilization of local ecosystem services are essential for food system resilience and for food security in parts of the world where high vulnerability and lack of coping capacity exist to combat climate change. Patterns of ecosystem services-based strategies were revealed that can be introduced to cope and adapt to climate-related natural hazards at the smallholder food system level. The study suggests that food system diversification, technological innovations and nature-based practices, and traditional and indigenous knowledge operationalized across the food system components have a potential for sustaining smallholder resilience in the face of natural hazards.

## 1. Introduction

There is a paucity of research that describes the linkages between ecosystem services, food systems, and food security for small-land owners and fisheries. Furthermore, how food systems can be more resilient has become more important on the list research agenda items in the time of amplitudinous natural hazards. The imminent loss or damage to agriculture, livestock, fishery, and forestry originating from nature is compounded by global and local market distortions, political and social upheavals that constrains the resiliency of food systems, and their ability to deliver safe and sufficient food. At the same time, enhancing food systems can decrease poverty by creating jobs and sustainable livelihoods [1,2,3].

Natural hazards significantly impact the agri-food sector, leading to decreased crop yields and livestock productivity, as well as declines in fisheries and agroforestry in areas already vulnerable to food insecurity. Subsequently, these hazards affect food diversity, the nutrient density of foods, food safety, and food prices [4,5].

The Food and Agricultural Organization (FAO) determined that the cost of natural disasters, between 2008 and 2018, on the agricultural sector of low and lower-middle income countries was greater than USD 108 billion due to damaged or lost crop and livestock production. This cost was particularly detrimental to the livelihoods of smallholder and subsistence farmers, pastoralists, and fishers [6].

The literature on food systems resilience has generally concentrated on the responses of food systems to climate change [7,8,9,10]; natural hazards [11,12]; socioeconomic crises, e.g., due to the COVID-19 pandemic [13,14,15,16,17,18]; and implications for interventions to build resilience [19,20,21,22]. There is a growing number of studies on food system resilience in rural and marginalized communities and neglected territories that focus on the role of natural resources [23,24,25]. Most of the studies on food systems resilience have focused on the measurement or assessment of livelihood, community resilience [26,27,28,29,30,31], determinants of livelihood resilience [20,32,33], and the linkages between sustainable agricultural practices and resilience [33,34,35]. However, less attention has been given to the functional properties of ecosystem services and their contribution in offsetting the effects of climate-related natural hazards at the smallholder food system level.

There is a growing interest in promoting ecosystem services-based responses to climate-related natural disasters that benefit smallholder farmers [36,37,38]. As an example, forests contribute to resilience through the structural defense against wind and soil erosion, water regulation, and provision of timber and non-timber forest products [39]. Coastal plants, such as mangrove, and salt marshes reduce tidal bore and erosion from storms and inflowing tides, while diminishing the saltwater intrusion and sediment deposition with organic matter [40]. Leading-edge review papers provide comprehensive scientific-based evidence on the potential of ecosystems and their services to contribute globally to disaster risk reduction and climate change adaptation [40,41,42,43].

At the local level, smallholder farmers’ ability to utilize ecosystem services in and around farms helps them balance ecological and socioeconomic trade-offs, and thus support local food systems to cope with adversities [39,44]. Many smallholders are already implementing practices that maintain the food system while ensuring the continued provision of ecosystem services on which their household’s food security, income, and well-being depend [45,46,47,48,49]. The implementation of these practices results in a higher capacity of their food system to resist, cope with, and/or recover from extreme events [50,51]. However, syntheses of these experiences are still lacking.

In response, we provide an analysis and descriptive details of case studies as examples of ways in which provisioning, regulation and maintenance, and cultural ecosystem services are being embedded in coping strategies across selected smallholder food systems located mainly in the tropical and subtropical regions. These regions have a significant number of small farms that contribute substantially to the production of most food commodities and experience the highest frequency of natural hazards related to climate change [6,52]. These selected case studies are used to illustrate promising approaches, lessons learned, and remaining challenges on how to increase the resilience of agri-food systems abetting the benefits provided by nature. Thus, the aim of this article is to demonstrate how ecosystem services can affect the food security of smallholder farmers when exposed to climate-related natural hazards by increasing the resiliency of food systems.

## 2. Materials and Methods

This narrative review is based on published studies in English between 2005 and 2021 given the relatively recent emergence and consolidation of ‘ecosystem services’ and ‘food system resilience’ as a defined research area. The timeframe was determined based on the landmark Millennium Ecosystem Assessment (MEA), being a milestone in ecosystem services research, summarizing past ecosystem change and assessing the future of human well-being [53]. The inclusion of this period captures the most recent literature in the research field, reflects current trends in resilience knowledge, and provides useful and applicable sources of some important case studies of food system resilience in the context of natural hazards. All titles and abstracts were screened by one author (Y.V.) against the study PI(E)COS (population, intervention/exposure, comparison, outcome, and study design) criteria to ensure eligibility for inclusion (see Table S1). If it was unclear from the abstract alone whether an article was eligible for inclusion, the full text was reviewed. The bibliographies of the included studies were also screened for additional articles.

The ScienceDirect, Semantic Scholar, and SAGE databases were used as the primary sources to identify publications (see Figure S1). The following syntax was used for this search: TITLE-ABS-KEY ‘food system(s) resilience’ AND TITLE ‘ecosystem services’ AND ‘natural hazards’ or ‘shocks’. Two researchers (Y.V. and D.T.) independently reviewed titles. Abstracts identified from the literature searches and reference list checking were imported to EndNote, and duplicates were removed. The titles and abstracts of all articles retrieved were independently screened for inclusion. In case of disagreement, consensus on which articles to screen full-text was reached by discussion. Next, Y.V. and D.T. independently screened full-text articles for inclusion. Disagreements or uncertainty about eligibility were resolved through discussion based on the study design and analysis methods.

Accordingly, we reviewed the scientific literature on the relationship between ecosystem services and food systems, and identified case studies as examples for how ecosystem services support small-scale subsistence farmers, pastoralist, and fishers with low productivity. Cases include factors related to shocks and stresses triggered by flooding, landslides, windstorms, droughts, extreme temperatures, and wildfires. The selected case studies allow tracking ecosystem services-based initiatives that are deemed to improve the capacity of smallholders to cope with extreme events, thus revealing both the interventions implemented and the outcomes generated.

## 3. Results and Discussion

### 3.1. Ecosystem Services Implications for Food System Resilience

Resilience thinking has its origins in ecology [54]. However, resilience thinking has been increasingly adopted as a generic approach to the behavioral understanding of social-ecological systems, which comprise a nexus of food production, processing, distribution, consumption systems, and ecosystem services [55,56].

Ecosystem services are the ecological characteristics, functions or processes that directly or indirectly contribute to human wellbeing: that is, the benefits that people derive from functioning ecosystems [53,57]. According to Common International Classification of Ecosystem Services (CICES) [57] provisioning services are the material and energetic outputs from ecosystems from which goods and products are derived. The regulation and maintenance category includes all the ways in which ecosystems can mediate the environment in which people live or depend on in some way, and benefit from them in terms of their health or security, for example. Finally, the cultural services category identified all the non-material characteristics of ecosystems that contribute to mental and intellectual well-being. In the context of ecosystem services, resilience is defined as the capacity of socio-ecological systems to continue providing a desired set of ecosystem service flows in the face of unexpected shocks and ongoing changes [58].

According to Tendall et al. [59], food system resilience is the capacity over time of a food system and its units at multiple levels to provide sufficient, appropriate, and accessible food to all in the face of various and unforeseen disturbances. In this context, “sufficient” means having enough food of nutritional quality; “appropriate” addresses food that is culturally, technically, and nutritionally appropriate; and “accessible” means that the food is physically and economically available to the population. These components represent the FAO dimensions of food security: availability, access, and utilization [60]. Whereas sustainability can be seen as an overall goal and has implications for food security for future generations, resilience is a means to achieve sustainability during change and disturbances [59]. Yet, the concept of food system resilience is less well-studied than food systems sustainability [34,55,61].

Food system resilience is closely related to ecosystem services. As climate disturbances increasingly lead to crop failures from floods, droughts, and disease, they are often exacerbated by an insufficient support for regulating ecosystem services. For example, vegetation cover is one of the factors that determines the risk and intensity of flash floods as it influences the water runoff as a natural process. The ecosystem function in this case is water flow regulation (slowing the passage of surface water). If people derive a benefit from this function, then that ecosystem function is regarded as a service (flash flood prevention and control). The main beneficiaries of this ecosystem service are different lowland communities that profit from a mitigation of flood peaks. The resilience value of this service can be described as the protection of livelihoods and can be defined, for example, by the calculation of post-flood biomass yield increment in terms of crop production.

The general consensus on the characteristics of the resilient social-ecological system include [62,63,64]: good ecological health, heterogeneity in ecological composition, and social livelihoods (diversity of properties, and activities, pathways, and flexibility to nimbly move between these [65]; resource use efficiency [66]; the existence of opportunities, resources, and information for learning; the acquisition of skills and knowledge by society [67]; effective institutions [68]; and social cohesion and the capacity and autonomy for democratic self-organization [69]).

Although all food systems have the same essential attributes, they vary significantly in different regions of the world and hence have different interactions with natural resources. How natural resources underpin all ecosystem activities and how these activities impact natural resources vary considerably from case to case [70,71]. This implies that the availability and variability of ecosystem services can be determinants of food systems resilience. We summarized these key factors into a framework that distinguishes the different aspects of resilience (see Table S2).

Five capacities of resilient food systems to manage climate changes and shocks to the ecosystem are considered essential [72,73]. These include the capacity to (i) anticipate the need for new approaches, (ii) prevent the impact of shocks, (iii) absorb the shocks for maintaining a functioning food system, (iv) adapt to an evolving risk, and (v) transform the current food system to make it sustainable [11]. These capacities for resilience require many of the same qualities of social and natural systems [74]. The definitions of these capacities with examples of delivered ecosystem services are given in Table 1.

The capacity of ecosystem services to contribute to food system resilience under external disturbances [77,78], such as extreme weather conditions, has been previously described and presented in Supplementary Materials (see Table S3).

Determining the valuation of ecosystem services (by amount, type, source, etc.) is essential to scale food system resilience and inform resilience policy. Mafongoya et al. developed indicators to identify and quantify ecosystem services in relation to food system sustainability and resilience, focusing on the group and classes of ecosystem services. [79]. In addition, several multiservice assessment models have been developed to provide accessible, quantitative assessments of ecosystem services (e.g., InVEST [80], LUCI [81], and ARIES [82]). However, while promising, due to limited available data, these models do not comprehensively characterize cultural ecosystem services that support the health and well-being of people having access to them nor reduce their susceptibility to experience harm [83]. It is an evidence-based concept that direct contact with nature supports the mental and physical health of people, including the spiritual experience and sense of place within societies [84,85]. This service provided by ecosystems is particularly important considering the social and health impacts of natural hazards.

The above analyses show that all categories of ecosystem services and their functional properties are relevant for resilience through advancing coping and adaptive capacities. The provisioning services mainly reduce the vulnerability of smallholder farmers by ensuring food and access to safe drinking water, raw materials, and medicinal resources [86,87]. A global study on the economic contribution of wild foods to rural livelihood revealed that 77 percent of households harvest wild food, which represents an important source of income generation and food security [88]. The regulation and maintenance of ecosystem services help to buffer the disaster-related impacts using mechanisms of water purification and waste treatment, air quality maintenance, soil erosion control, flood protection, climate regulation, pest and disease regulation, pollination, and regulation of the frequency and intensity of natural hazards’ flow while enabling the richness of provisioning services [89,90]. Cultural ecosystem services are considered to support the health and well-being of people having access to them and, with this, reduce their susceptibility to experience harm [40,91,92]. As an example, an Indigenous Fijian community revealed a strong sense of belonging and social identity with the land, river, and ocean. The use of ecosystem services is not only vital for maintaining livelihood security but is equally important to the existence of their cultural identity, helping villagers adapt to the adverse impacts of climate change [93].

Despite the comprehensive studies on ecosystem services and their functions in recent years, there remains a lack of practical understanding about the variety of stakeholders, their food system vulnerabilities, and perceptions and preferences over different types of ecosystem services against climate change-induced natural hazards, especially in natural and semi-natural rural areas [94,95,96]. The following section illustrates examples of how resilience interventions are prioritized based on existing agri-food activities and available ecosystem services.

### 3.2. Empirical Illustrations of Integrating Ecosystem Services to Advance the Smallholder Farmer Food Systems' Resilience

Having a resilient food system enhances the capacity to respond to adverse events [15,97,98]. One of the major strategies to improve the resilience of food systems is to transform current input-intensive, monoculture-dominated cropping systems into temporally, spatially, and functionally diversified agroecosystems [78,98,99,100]. However, this transition must be context dependent [29,101] and may not be transferrable across sectors, since most analysis and assessments have compared resilience in the same country or region with similar natural, institutional, and socioeconomic contexts [102,103] A screening between different types of food systems, ecosystems, and climate-related natural hazards would help in the understanding of the resilience prerequisites and reveal patterns of ecosystem services-based strategies that can be introduced to cope and adapt to climate change. Thus, we explored the influence of the socioeconomic and natural context on the smallholder food system resilience to distinguish types of interventions. To maintain the level of detail of information contained in several case studies and allow for generalizability, the empirical data were analyzed and structured according to several predefined themes within the resilience framework: study location, principal food systems, and related ecosystems services; natural hazards; and the choice of resilience interventions and its subsequent or anticipated effect on the food system/farm/region (see Table S4).

Whereas the case studies outlined in Table S3 differ in their research focus and methodological approaches, there were patterns about the impacts of nature-related shocks and stresses on food systems and implemented resilience strategies. Nature-related extremes considered in these examples ranged from major drought and flood events to devastating storms across the word. These studies identified that the most resilience strategies were formed and applied using local knowledge and experience in addition to formal and informal extension networks [104,105].

The resilience strategies have been classified by authors into three main categories based on patterns that often include similar and overlapping components and have resilience outcomes: (i) promoting food system diversification, (ii) increasing cohesion between technological innovations and nature-based solutions, (iii) utilizing traditional and local/indigenous knowledge. Each category is presented below, along with the interlinkages between available ecosystem services and their implications to resilience strategies.

#### 3.2.1. Promoting Food System Diversification

Relying on a ‘mono’, e.g., monocropping, mono diet, or mono production, is considered risky for many farmers. Any change in the environment—be it drought, erratic rainfall, or a temporary frost—can undermine food system sustainability. Thus, farmers who value resilience often minimize their risks by expanding and diversifying their chains in the food system: adding new crops and products; making use of their farms for non-agricultural activities; making integrations, including integrated aquaculture with polyculture farming of multiple species such as shrimp, mud crab, milkfish, seaweed, and bivalves [106]; and using crop/aquaculture and crop-livestock integration [107,108].

Through an integrated relationship between crop and livestock components, farmers can increase agricultural productivity per unit of land and water beyond the productivity of the individual components [109]. For instance, the precarious nature of rubber is experienced by smallholders around the world, as the interval time of approximately 7 years between planting and harvest can be difficult for smallholders to recover, and climate change is likely to exacerbate the environmental marginalization of these plantations. Therefore, the choice of agricultural enterprise will influence household resilience [110]. Similarly, Davis et al. [111] found that the food security challenges facing coffee farmers can be alleviated by the improved marketing of fruit tree products under shade coffee farming systems to provide multiple co-benefits, including watershed management. These studies support the diversification of management strategies to keep highly managed food systems stable and productive, and to maintain biodiversity.

#### 3.2.2. Increasing Cohesion between Technological Innovations and Nature-Based Solutions

Modern challenges entice farmers to replace their traditional methods of production with more advanced technologies and integrate agricultural practices into ecosystem services. This strategy often requires an adjustment of the farming system at the farm and regional levels. For instance, integrating suitable cereal and legume varieties and livestock breeds with a combination of locally sourced and supplementary inputs can increase overall farm profitability and resilience [109]. Adjusting the seasonal calendar for crop production to avoid heavy rains and floods, using short-duration and tolerant crop varieties, and practicing mixed cropping to reduce the risk of total crop failure with climate-tolerant seeds is another example of how farmers can reduce risk by adapting farm practices.

Nature-based practices (e.g., making different types of terraces, planting nitrogen-fixing crops, planting trees, applying organic fertilizers, etc.) are a method to prevent net losses in crop yield and can be enhanced by the additional biomass produced from wild plants and animals. In Africa, the links between tree cover, access to food, and improved dietary diversity are also becoming increasingly evident [111,112]. Pandey et al. concluded that rainwater harvesting systems also functioned as a climate change adaptation strategy, contributing to resilience [113,114].

Examples are provided below to illustrate the capacity of ecosystem services for enhancing food systems’ resilience, reflecting real-life situations and trends.

Coffee is one of the most important commercial tropical crops in terms of its gross production value and role in livelihoods. Coffee production and quality are expected to be negatively affected by increases in temperature and changes in rainfall patterns. Although reduced coffee production may not threaten food security per se, local food systems depend on this crop. Nature-based solutions can improve coffee’s climate resilience, benefiting coffee farmers whose livelihoods depend on this crop. For example, diverse shade canopies provide nesting and foraging habitats for both bees and birds, increasing richness and abundance. Increasing crop and overall plant diversity within fields and field margins, edges, pathways, and live fences can benefit both bees and birds, since allowing non-crop plant and weed species to grow and flower on farms can provide forage resources to complement the brief and intense flushes of coffee flowers themselves. As an example, birds lowered infestation rates as much as 50% in simplified and intensively managed agroforestry coffee farms, and as much as 58% in sun coffee farms [115]. De Marco and Coelho (2004) estimated that, in Brazil, coffee close to native forests increased production value by USD 1860 ha/year [116]. Managing coffee farms based on wide ecosystem services application could improve the climate resilience of coffee cropping and communities of birds and bees, and therefore help farming families adapt their food systems to the changing environment.

Conservation and restoration are two additional pathways that smallholder farmers can support ecosystem services, hence supporting the resilience of food systems [38]. Conservation involves adopting practices that maintain ecosystem service and restoration for their recovery. Recent evidence has shown that pastoral householders in Northeast Ethiopia are more resilient to drought events than others due to the application of soil and water conservation techniques and access to appropriate irrigation [117]. Farmers in Papua New Guinea employ mulching as a soil management practice to support agri-food system resilience [62]. Similarly, potato farmers in Kenya change seed regimes, rotate crops, and engage in minimum tillage as knowledge-intensive conservation practices [33,118].

The establishment of protected areas can also affect local food systems, and can change how terrestrial and marine natural resources are used and managed with consequences to food system resilience. A case in Aventureiro Brazil (see Table S4) highlighted resilience activities due to tradeoffs among the community geographical location, conservation drivers, and development drivers. Livelihood diversification contributes to food system resilience by promoting lower dependence on natural resources for households and improves financial capital [119]. Even in situations when access to resources is very constrained, small-scale actors with an ability to improvise and hustle natural resources effectively in the short term [120], for instance, the conversion of the tidal flat to cropland, reforestation, and soil conservation practices, planting in shaded areas, etc., can cope with shocks or persistent stresses [121].

A study by Chowdhury et al. [122] on eco-engineered reefs focused on of the development of resilience in coastal regions that are home to nearly 2.5 billion people living within 62 mi of coastlines. These regions are especially vulnerable to habitat loss, sea-level rise, and other climate change effects. Oyster-dominated eco-engineered reefs have been promoted as integral components of engineered habitats, enhancing coastal resilience through the provision of numerous ecological, morphological, and socioeconomic services. Oysters not only serve as a highly valued food source but they also provide many critical ecosystem services, such as a habitat for many recreationally and commercially important fisheries, and water quality enhancement by filtering suspended material (or seston) from the overlying water column. Oysters also support the socioeconomic resilience of coastal communities through the provision of food, physical and mental health, and cultural values. Ecosystems services-based technological solutions have the potential to provide unique and invaluable tools for reducing vulnerability to natural hazards through added complexity, which generates a web of self-supporting and self-regulating interactions.

#### 3.2.3. Utilizing Traditional and Local/Indigenous Knowledge

A growing body of research illustrates that local people have significant resilience and actively observe and adapt to change in a diversity of ways [123,124,125]. An indigenous perspective helps to strengthen resilience capacities by placing greater emphasis on the value of broad local knowledge of the climate, ecology, and hazards, leading to a series of historical adaptations. For example, according to Campbell (2021), coffee farmers have utilized indigenous knowledge systems built on local ontology and cosmovision to sharpen their sensitivity to environmental conditions. They often monitor early warning signals for episodic events especially regarding changes in temperature and rainfall. In many cases, local knowledge is the only tool farmers have to negotiate multiple livelihood stressors [32].

Strong emotional reactions leading to ecological grief have been documented among Inuit because changing ice and weather regimes reduce access to traditional hunting and fishing locations [126], whereas some Inuit communities are transforming from land-based (e.g., caribou) to aquatic-based livelihoods to build resilience [117,124]. Peruvian Quechua-speaking people have confronted the impacts of colonization by creating the Potato Park to protect over 900 varieties of potato, reinvigorating cultural values of reciprocity, kinship, and solidarity that underpin community resilience [127]. The Bedamuni tribe of Papua New Guinea retain traditional knowledge regarding coping strategies to hazards, e.g., El Nino (droughts and fires), earthquakes, floods, pest and disease outbreaks, and strong winds. Tribe representatives cope with food system susceptibility by transplanting rhizomes from existing gardens to creeks, rivers, swamps, or caves during drought, or through the use of famine foods (e.g., bush yams, black palm shoots, insects, “poisonous nuts” and “soft rocks”) and the temporary migration of some villagers to dense forests less affected by drought to collect bush food [128]. Indigenous knowledge serves as guidance and entry points for increasing the resilience of local food systems, particularly of marginalized communities. Adhikari, Hussain, and Rasul (2017) noted the preference of farmers in the Rasuwa district, in the mid-hills of Nepal, for local bean, barley, millet, and maize, rather than commodity crops, because they are more tolerant to water stress and extremely cold conditions [129].

According to an FAO report, the way Indigenous people consider their relationship with the environment and human needs is unique [125]. Their food systems can exceed 250 wild, semi-domesticated, and domesticated plants, fish, and animals used for food and non-food purposes (medicinal remedies, construction materials, fuel, fodder, etc.) [130,131]. Territorial management practices incorporate a broad scope of livelihood activities, such as gathering, hunting, fishing, and farming adjusted to seasonal cycles and natural patterns observed in ecosystems [112,132]. Wind, water, and solar energy stored in firewood or other biomass meets the basic needs for food processing, heating, and cooking. The synergy of traditional practices increases the level of food self-sufficiency in the food systems oscillated from about 55 percent to about 80 percent [125], allowing Indigenous people to adapt and better cope with the impacts of natural hazards.

The preservation of indigenous knowledge of traditional farming is advantageous in maintaining biodiversity [133], enhancing food security [134,135], and protecting natural resources [136].

The examined case studies support the concept that empowering smallholder farmers to understand their local situation, and to identify appropriate adaptive strategies and needs based on ecosystem services, are essential to improving food systems resilience [93]. Food system diversification, technological innovations and nature-based practices, and traditional knowledge are essential prerequisites for increasing the smallholder food systems’ resilience to natural hazards.

## 4. Conclusions

The results of the narrative review offer a deeper understanding of the functional properties of ecosystem services and their contribution in coping with the effects of climate-related natural hazards at the smallholder food system level.

This paper extends the previous work by Carpenter et al., [137], Dipierri and Zikos [138], and Ostrom [139,140] by explicitly linking the role of ecosystem services to food security and ultimately the nutritional status of individuals and communities. Historically, the MEA and other works have focused on the how they relate to well-being without specific reference to food security [137]. We have extended their work to provide examples of how natural resources can also support smallholders without human interference, which allows ecosystem services support the adaptation and mitigation to climate change-induced natural hazards. This does not negate the fact that policies and the design principles articulated by Ostrom [140] do not ultimately impact the relationship between ecosystem services, food systems, and food security [138]. Other researchers have long investigated how and why managing ecosystem services may significantly contribute to resilient and sustainable food systems to the impacts of natural hazards. This paper contributes to the existing literature in two ways. First, we synthesized the functional properties of ecosystem services and their contribution in offsetting the effects of climate-related natural hazards from the smallholder food system perspective. Second, the study reveals ecosystem services-based strategies that can be utilized across different types of traditional smallholder food systems to cope and adapt with natural hazards. By combining the theoretical approaches with case examples, this paper contributes to a better understanding of what functional properties of ecosystems have the capacity to make food systems more resilient.

Empirical illustrations have revealed the significant barriers that smallholders withstand to maintain their livelihoods and food systems under natural hazards. The deteriorating quality of the natural resource base, reliance on rain-fed farming, abandonment of traditional varieties of crops for non-nutritive cash crops, nature conservation initiatives restricting access to wild food sources, declining productivity, low value-added activities, lack of infrastructure, and service provision are some of the most acute.

In these conditions, people put into action their shared knowledge about local nature and activated ecosystem services. The empirical illustrations suggest that smallholder farmers have a higher resilience capacity and maintain their food systems by ecosystem services utilization.

The proposed ecosystem services-based interventions provide advancement for smallholder farmers who use traditional and local/indigenous knowledge to promote food system diversification to increase cohesion between technological innovations and nature-based solutions. These activities are frequently used to increase food security and improve livelihood opportunities irrespective of the external shocks and stresses.

These findings highlight the importance that ecosystem services have on supporting and regulating food system resilience in relation to environmental and socioeconomic conditions. In the long term, additional studies must be conducted to expand ‘win-win’ ‘mutually beneficial’ solutions that support the resilience of both food systems and ecosystem services. More importantly, we emphasize that no single set of ecosystem services-based interventions can be followed in the design of food system resilience management across the world. Rather, there is a need to consider homegrown and unique characteristics of nature in conjunction with food system patterns to co-design resilient frameworks suited for local communities. An ecosystem services approach can be used for the ex-post analysis of food system dynamics and responses to challenges, and for the ex-ante assessment and creation of resilience-enhancing strategies and attributes of food systems [141].

These results underscore the potential that ecosystem services must enhance the resilience capacity of the smallholder food systems to natural hazards. To reap benefits from ecosystem services, future resilience stewardship must increase structural heterogeneity and diversity across multiple dimensions, including landscapes, food provision, and consumption activity.

## Figures and Tables

**Table 1:** Ecosystem services contribution to food systems resilience.

Capacity	Definition	Ecosystem Services Implications	Examples of Contributions to Food System Resilience	Related Indicators for ES Delivered
Anticipate	The capacity in creating systems that can maintain its state in response to the unexpected crises.	Lifecycle maintenance, habitat, and gene pool protection; pest and disease control; regulation of soil quality; water conditions; atmospheric composition and conditions.	Pollination and seed dispersal; weathering processes and their effect on soil quality; regulation of the chemical condition of water by living processes; micro and regional climate regulation.	Pollinators’ species richness; host-species (trees); abundance number of beehives, areal coverage of vegetation (hedgerows, flower strips, high nature farmland); soil organic matter content; carbon sequestered; humidity index.
Prevent	The capacity of a system and its properties to cushion against stresses and shocks.	Regulation of baseline flows and extreme events.	Buffering and attenuation of mass movement; control of erosion; hydrological cycle and water flow regulation—flood control and coastal protection; wind protection, fire protection.	Density of hedgerows; percentage of soil cover; soil erosion risk; retention capacity of water in agricultural soils; share of agroforestry within floodplains.
Absorb	The capacity of change that a system can undergo while still retaining its function and structure.	Mediation of wastes or toxic substances of anthropogenic origin by living and non-living processes).	Bioremediation, filtration/sequestration/storage/accumulation by micro-organisms, algae, plants, and animals; dilution by atmosphere, freshwater and marine ecosystems; smell reduction; visual screening.	Concentration of pollutants in soil in agricultural areas; hedgerow length.
Adapt	The capacity of learning, combining experience and knowledge, and adjustments to external drivers.	Cultivated terrestrial and aquatic plants for nutrition, materials, or energy; reared animals for nutrition, materials or energy e.g., aquatic; wild plants and animals; genetic material from plants, algae, fungi, animals; water used for nutrition, materials or energy.	Increased food and nutrition security and profits; use of agro-ecologically suitable high yielding varieties; greater food diversity of diets and micronutrient.	Yields of food and feed crops; livestock data; wild game population estimates; energy from manure treatment systems; volume of water bodies.
Transform	The creation of a new system through making a fundamental change of its characteristics and actions when the initial state is not bearable anymore.	Mineral/non-mineral substances or ecosystem properties used for nutrition, energy (wind, solar, geothermal); physical, experiential, intellectual, and representative interactions with natural environment.	Diversified income sources buffer against climate fluctuations; switch to renewable energies, curbing demand for high energy use technologies.	Number of hunting licenses; number of scientific studies on agro-ecosystems; cropland or grassland in protected agricultural areas; alternative energy production.

Based on [21,75,76].

## Supplementary Materials

The following supporting information can be downloaded at: https://www.mdpi.com/article/10.3390/ijerph19063652/s1, Figure S1: PRISMA chart showing study number at each stage; Table S1: PICOS criteria for inclusion and exclusion of studies; Table S2: Key aspects of resilience and their implementation in the study; Table S3: The interaction between the food system and ecosystem services; Table S4: Natural hazards and food systems’ resilience interventions.
