# Supplementary material for: Considering Ecosystem Services in Food System Resilience
Source: Int J Environ Res Public Health. 2022 Mar 19;19(6):3652. doi: 10.3390/ijerph19063652 (PMC8954919; doi:10.3390/ijerph19063652)
Supplement: Supplementary file 1 [file ijerph-19-03652-s001.zip › Table S1. PICOS_criteria.pdf]

**Table S1.** PICOS criteria for inclusion and exclusion of studies

| Parameter | Inclusion criteria | Exclusion criteria |
|---|---|---|
| General | Published in English | Foreign language studies |
| | Published between 2005 and 2021 | Unpublished studies |
| Study design | Relevant systematic reviews, or meta-analyses (used for identifying additional studies) | Non-original data (e.g. narrative reviews, editorials, letters) |
| | Case study | Experimental research |
| | Descriptive research | Causality studies |
| | Observational study | Sequential research |
| | Mixed-Methods research | Philosophical analysis |
| | Field research | Historical research |
| | Exploratory research | Government documents |
| | Action research | Diagnostic research |
| | Cross-sectional research | Methodological or technical documents that reported only the methodology and methods |
| | Insight reports | Theoretical studies |
| Population | Population of local rural and suburban areas as a primary. No limitation was set for age groups | Population of urban areas |
| Exposure/intervention | Natural hazards, hydrometeorological hazards as a primary | Studies that focus solely on ecosystems, ecosystem services, food systems, natural hazards |
| | | Description of resilience measures that do not clearly align with ecosystem services |
| Setting | Local food systems (households, communities, districts) of rural and suburban areas as a primary | Global, national and regional food systems of urban areas |
| Outcomes | The challenges faced by the food systems to natural hazards and ecosystem services contribution to meet these challenges | Socio-economic disruptions of the food supply operations leading to food shortage, food losses, or price volatility in both rural and urban areas |
