# Supplementary material for: Considering Ecosystem Services in Food System Resilience
Source: Int J Environ Res Public Health. 2022 Mar 19;19(6):3652. doi: 10.3390/ijerph19063652 (PMC8954919; doi:10.3390/ijerph19063652)
Supplement: Supplementary file 1 [file ijerph-19-03652-s001.zip › Table S2_Key_aspects_resilience.pdf]

Table S2. Key aspects of resilience and their implementations in the study

Aspects of resilience	Definition	Implementation in the study	Terminology
'Resilience to'	Drivers/triggers that may disrupt the system	Natural hazards	Natural hazards are predominantly associated with natural processes and phenomena. They include biological, environmental, geological, and hydrometeorological processes and phenomena [59].
'Resilience of'	Function and identity of the system	Food system	Food system encompass the entire range of actors and their interlinked value-adding activities involved in the production, aggregation, processing, distribution, consumption and disposal of food products that originate from agriculture, forestry or fisheries, and parts of the broader economic, societal and natural environments in which they are embedded [60].
'Resilience at'	Scales at which the system is observed	Smallholder	Smallholder food systems comprise small-scale farms, pastorals, forestry, fishery on areas varying from less than one hectare to 10 hectares. Smallholder food systems are characterized by using mainly family labor for production and using part of the produce for family consumption [61].
'Resilience due to'	System characteristics that potentially cause resilience	Ecosystem services	Ecosystem services (ES) are the ecological characteristics, functions or processes that directly or indirectly contribute to human wellbeing: that is, the benefits that people derive from functioning ecosystems [39, 44].

Based on: Bennett et al. (2005), Carpenter et al. (2001), Chen et al. (2019), Authors.
