# Supplementary material for: Considering Ecosystem Services in Food System Resilience
Source: Int J Environ Res Public Health. 2022 Mar 19;19(6):3652. doi: 10.3390/ijerph19063652 (PMC8954919; doi:10.3390/ijerph19063652)
Supplement: Supplementary file 1 [file ijerph-19-03652-s001.zip › Table S3_ES_FS_interaction.pdf]

pTable S3. Interaction between the food system's elements and ecosystem services

Ecosystem Services Classification (CICES V5.1)				Food System Elements					
**Section**	**Division**	**Group**	**Class**	**1**	**2**	**3**	**4**	**5**	**6**
**Provisioning (Biotic)**	Biomass	Cultivated terrestrial plants for nutrition, materials or energy	Cultivated terrestrial plants (including fungi, algae) grown for nutritional purposes						
			Fibers and other materials from cultivated plants, fungi, algae and bacteria for direct use or processing (excluding genetic materials)						
			Cultivated plants (including fungi, algae) grown as a source of energy						
		Cultivated aquatic plants for nutrition, materials or energy	Plants cultivated by in- situ aquaculture grown for nutritional purposes						
			Fibers and other materials from in-situ aquaculture for direct use or processing (excluding genetic materials)						
			Plants cultivated by in- situ aquaculture grown as an energy source						
		Reared animals for nutrition, materials or energy	Animals reared for nutritional purposes						
			Fibers and other materials from reared animals for direct use or processing (excluding genetic materials)						
			Animals reared to provide energy (including mechanical)						
		Reared aquatic animals for nutrition, materials or energy	Animals reared by in-situ aquaculture for nutritional purposes						
			Fibers and other materials from animals grown by in-situ aquaculture for direct use or processing (excluding genetic materials)						
			Animals reared by in-situ aquaculture as an energy source						
		Wild plants (terrestrial and aquatic) for nutrition, materials or energy	Wild plants (terrestrial and aquatic, including fungi, algae) used for nutrition						
			Fibers and other materials from wild plants for direct use or processing (excluding genetic materials)						
			Wild plants (terrestrial and aquatic, including fungi, algae) used as a source of energy						

Ecosystem Services Classification (CICES V5.1)				Food System Elements					
Section	Division	Group	Class	1	2	3	4	5	6
		Wild animals (terrestrial and aquatic) for nutrition, materials or energy	Wild animals (terrestrial and aquatic) used for nutritional purposes						
			Fibers and other materials from wild animals for direct use or processing (excluding genetic materials)						
			Wild animals (terrestrial and aquatic) used as a source of energy						
	Genetic material from all biota (including seed, spore or gamete production)	Genetic material from plants, algae or fungi	Seeds, spores and other plant materials collected for maintaining or establishing a population						
			Higher and lower plants (whole organisms) used to breed new strains or varieties						
			Individual genes extracted from higher and lower plants for the design and construction of new biological entities						
		Genetic material from animals	Animal material collected for the purposes of maintaining or establishing a population						
			Animal material collected for the purposes of maintaining or establishing a population						
			Wild animals (whole organisms) used to breed new strains or varieties						
		Genetic material from organisms	Individual genes extracted from organisms for the design and construction of new biological entities						
**Provisioning (Abiotic)**	Water	Surface water used for nutrition, materials or energy	Surface water for drinking						
			Surface water used as a material (non-drinking purposes)						
			Freshwater surface water used as an energy source						
			Coastal and marine water used as energy source						
		Ground water for used for nutrition, materials or energy	Ground (and subsurface) water for drinking						
			Ground water (and subsurface) used as a material (non-drinking purposes)						
			Ground water (and subsurface) used as an energy source						
	Non-aqueous natural	Mineral substances used for nutrition,	Mineral substances used for nutritional purposes						
			Mineral substances used for material purposes						
			Mineral substances used for as an energy source						

Ecosystem Services Classification (CICES V5.1)				Food System Elements					
**Section**	**Division**	**Group**	**Class**	**1**	**2**	**3**	**4**	**5**	**6**
		materials or energy							
		Non-mineral substances or ecosystem properties used for nutrition, materials or energy	Non-mineral substances or ecosystem properties used for nutritional purposes						
			Non-mineral substances used for materials						
			Wind energy						
			Solar energy						
			Geothermal						
**Regulation & Maintenance (Biotic)**	Transformation of biochemical or physical inputs to ecosystems	Mediation of wastes or toxic substances of anthropogenic origin by living processes	Bio-remediation by micro-organisms, algae, plants, and animals						
			Filtration/sequestration/storage/accumulation by micro-organisms, algae, plants, and animals						
		Mediation of nuisances of anthropogenic origin	Smell reduction						
			Noise attenuation						
			Visual screening						
	Regulation of physical, chemical, biological conditions	Regulation of baseline flows and extreme events	Control of erosion rates						
			Buffering and attenuation of mass movement						
			Hydrological cycle and water flow regulation (Including flood control, and coastal protection)						
			Wind protection						
			Fire protection						
		Lifecycle maintenance, habitat and gene pool protection	Pollination (or 'gamete' dispersal in a marine context)						
			Seed dispersal						
			Maintaining nursery populations and habitats (Including gene pool protection)						
		Pest and disease control	Pest control (including invasive species)						
			Disease control						
			Weathering processes and their effect on soil quality						

**Ecosystem Services Classification (CICES V5.1)**				**Food System Elements**					
**Section**	**Division**	**Group**	**Class**	**1**	**2**	**3**	**4**	**5**	**6**
		Regulation of soil quality	Decomposition and fixing processes and their effect on soil quality						
		Water conditions	Regulation of the chemical condition of freshwaters by living processes						
			Regulation of the chemical condition of salt waters by living processes						
		Atmospheric composition and conditions	Regulation of chemical composition of atmosphere and oceans						
			Regulation of temperature and humidity, including ventilation and transpiration						
**Regulation & Maintenance (Abiotic)**	Transformation of biochemical or physical inputs to ecosystems	Mediation of waste, toxics and other nuisances by non-living processes	Dilution by freshwater and marine ecosystems						
			Dilution by atmosphere						
			Mediation by other chemical or physical means (e.g. via Filtration, sequestration, storage or accumulation)						
		Mediation of nuisances of anthropogenic origin	Mediation of nuisances by abiotic structures or processes						
	Regulation of physical, chemical, biological conditions	Regulation of baseline flows and extreme events	Mass flows						
			Liquid flows						
			Gaseous flows						
		Maintenance of physical, chemical, abiotic conditions	Maintenance and regulation by inorganic natural chemical and physical processes						
**Cultural (Biotic)**	Direct, in-situ and outdoor interactions with living systems that	Physical and experiential interactions with natural environment	Characteristics of living systems that that enable activities promoting health, recuperation or enjoyment through active or immersive interactions						
			Characteristics of living systems that enable activities promoting health, recuperation or enjoyment through passive or observational interactions						

Ecosystem Services Classification (CICES V5.1)				Food System Elements					
**Section**	**Division**	**Group**	**Class**	**1**	**2**	**3**	**4**	**5**	**6**
		Intellectual and representative interactions with natural environment	Characteristics of living systems that enable scientific investigation or the creation of traditional ecological knowledge						
			Characteristics of living systems that enable education and training						
			Characteristics of living systems that are resonant in terms of culture or heritage						
			Characteristics of living systems that enable aesthetic experiences						
	Indirect, remote, often indoor interactions with living systems that do not require presence in the	Spiritual, symbolic and other interactions with natural environment	Elements of living systems that have symbolic meaning						
			Elements of living systems that have sacred or religious meaning						
			Elements of living systems used for entertainment or representation						
		Other biotic characteristics that have a non-use value	Characteristics or features of living systems that have an existence value						
			Characteristics or features of living systems that have an option or bequest value						
**Cultural (Abiotic)**	Direct, in-situ and outdoor interactions with natural physical systems that depend on presence in the environmental setting	Physical and experiential interactions with natural abiotic components of the environment	Natural, abiotic characteristics of nature that enable active or passive physical and experiential interactions						
		Intellectual and representative interactions with abiotic components of the natural environment	Natural, abiotic characteristics of nature that enable intellectual interactions						

Ecosystem Services Classification (CICES V5.1)				Food System Elements					
**Section**	**Division**	**Group**	**Class**	**1**	**2**	**3**	**4**	**5**	**6**
	Indirect, remote, often indoor interactions with physical systems that do not require presence in the environmental setting	Spiritual, symbolic and other interactions with the abiotic components of the natural environment	Natural, abiotic characteristics of nature that enable spiritual, symbolic and other interactions						
		Other abiotic characteristics that have a non-use value	Natural, abiotic characteristics or features of nature that have either an existence, option or bequest value						

Note: Darker shaded cells indicate more obvious and relevant linkages that lighter colored cells, although this does not necessarily mean that they have no direct or indirect linkage with food system elements and ecosystem services; 1 to 6 are the components of food system: 1 – inputs; 2 – production; 3 – harvest and processing; 4 – post-harvest storage and transportation; 5 – retailing; 6 – cooking and consumption.
