# Supplementary material for: Considering Ecosystem Services in Food System Resilience
Source: Int J Environ Res Public Health. 2022 Mar 19;19(6):3652. doi: 10.3390/ijerph19063652 (PMC8954919; doi:10.3390/ijerph19063652)
Supplement: Supplementary file 1 [file ijerph-19-03652-s001.zip › Table S4_Challenges_ES_Interventions.pdf]

Table S4. Challenges and food systems' resilience interventions based on ecosystem services

No.	Authors	Year	Study Title	Study Location	Target food system	Backbone ecosystem services (ES)	Natural hazards	Consequences to food systems	Resilience Interventions based on ES	Outcomes
1	Islam MA, Paull DJ, Griffin AL, Murshed S. (Islam, Paull, Griffin, & Murshed, 2020)	2020	Assessing ecosystem resilience to a tropical cyclone based on ecosystem service supply proficiency using geospatial techniques and social responses in coastal Bangladesh	Kalapara, southwestern coastal region of Bangladesh. The area is diverse landcover with great range of geomorphological environments, including both coastal and fluvial features. Landcover types: cropland, riparian forest, mangrove forest, sandy	Cropland occupies more than half of the study area and agriculture is the main livelihood. Aquaculture practices, such as shrimp farming, are common within croplands. Livestock rearing on or adjacent to cropland, and crop residues satisfy fodder	Mangrove and riparian forests serve as windbreaks to protect local settlements and reduce storm surge destruction during cyclones. Dense forest improves air quality. Sandy beaches and mangrove forest help to promote	Tropical cyclones (Cyclone Sidr, 2007)	Cyclone triggered breaching of coastal and river embankments, resulted in inundation of vast areas of croplands in low-lying areas. The wind also caused widespread damage to vegetation and uprooted trees. Approximately ~ 45% of cropland (paddy)	Extensive planting of mangroves by the local forestry department after the cyclone. Few attempts to establish plantations of riparian forest. The conversion of most of the tidal flat to cropland.	Cropland, being the highest contributor to ecosystem services in this area, had recovered the most, demonstrating the highest resilience (94% had returned to their pre-cyclone state), followed by dense mangrove forest (73%),

No.	Authors	Year	Study Title	Study Location	Target food system	Backbone ecosystem services (ES)	Natural hazards	Consequences to food systems	Resilience Interventions based on ES	Outcomes
				beach and tidal flat.	needs after harvest.	the tourism sector. Numerous tidal flats are used for rearing poultry and livestock, especially ducks and buffalo.		and ~ 80% of trees were severely affected by strong winds and torrential rainfall. Livestock and aquaculture were seriously affected by the cyclone, negatively impacting local livelihoods.		sparse riparian forest (71%), tidal flat (62%), dense riparian forest (52%), sparse mangrove (44%), and sandy beach (42 %).
2	Melketo T, Schmidt M, Bonatti M, Sieber S, Müller K, Lana M. (Melketo et al., 2021)	2021	Determinants of pastoral household resilience to food insecurity in Afar region, northeast Ethiopia	Mille district of the Afar Region in Ethiopia, the arid and semi-arid	Pastoralist food system. Livestock satisfy household food consumpti	Reared animals for nutrition; limited surface water used for	Climate change-induced drought events (1973/74, 1983/84, 1991/92,	Drought events push dry land systems to cross biophysical	Livestock diversification is an advantage and a means for households to build	

No.	Authors	Year	Study Title	Study Location	Target food system	Backbone ecosystem services (ES)	Natural hazards	Consequences to food systems	Resilience Interventions based on ES	Outcomes
				lowland areas of the country, an area is prone to rainfall variability and extreme drought.	on demand, income security, and transportation. Since goats and camels are drought resistant, they are dominant and comprise prominent parts of the herd composition in the arid environment of Mille, which has shortages of water and grazing lands.	nutrition; limited arable lands	1999/00, 2005/06)	thresholds, causing a long-term drop in livestock productivity. With the deteriorating quality of the natural resource base, declining productivity, and falling animal per capita, pastoralists are unable to subsist on livestock and achieve food security, (77%)	resilience to shocks and stresses. Better resilient households are those that tend to diversify their animal husbandry mixes in response to the weather uncertainties and shocks. The household use of soil and water conservation techniques and its access to irrigation, significantly influencing	

No.	Authors	Year	Study Title	Study Location	Target food system	Backbone ecosystem services (ES)	Natural hazards	Consequences to food systems	Resilience Interventions based on ES	Outcomes
								reportedly becoming food insecure for more than five months per year.	the resilience status of pastoral households to food insecurity.	
3	Whitfield S, Beauchamp E, Boyd DS, Burslem D, Byg A, Colledge F, et al. (Whitfield et al., 2019)	2019	Exploring temporality in socio-ecological resilience through experiences of the 2015–16 El Niño across the Tropics	Mangrove-lagoon system, covering an area of 800km^2 on the Colombian Caribbean coast	Small-scale fisheries	Provision of fish, mollusks and crustacean catch; drinking water	Droughts and floods caused by El Nino events (2015-2016)	Changes in fishing practices due to variations in total fish, mollusks (sediments covered the natural oyster), crustacean catch, decline or loss of income-generating capacity, higher competitio	Greater reliance on dried rather than fresh fish. Elevation of agricultural fields and houses.	The system can continue to deliver consistent fishery benefits, and functioning markets, in the face of the combined effects of environmental change and

No.	Authors	Year	Study Title	Study Location	Target food system	Backbone ecosystem services (ES)	Natural hazards	Consequences to food systems	Resilience Interventions based on ES	Outcomes
								n for natural resources, scarcity of food and drinking water, concern over the sanitary quality of the food.		anthropogenic pressure demonstrates an apparent resilience of food system.
				Halaba district, Southern Ethiopia	Small-scale rain-fed maize agriculture and some cash cropping, teff and pepper. Livestock is limited to a few heads of cattle.	Surface and ground water; mineral substances (soil) for agricultural purposes.	Drought (2015), flood events following drought.	Serious impact on crop productivity and water availability and quality. Loss of livestock through death (feed shortages) or sale resulted not only in	Reforestation and soil conservation practices. Farmers relied on water sources (rivers), often unimproved and unsafe; correspondingly the time taken to collect water	Noticeable impacts of the drought were relatively spatially uniform. The subsequent flood impacts were highly localised. Experiences of acute

No.	Authors	Year	Study Title	Study Location	Target food system	Backbone ecosystem services (ES)	Natural hazards	Consequences to food systems	Resilience Interventions based on ES	Outcomes
								the immediate loss of income and food sources but also lowered households' resilience against future shocks.	increased due scarcity. Farmers sold livestock (at low prices) to purchase food.	damage to property and crops and long-term impacts on soil fertility, due to erosion and/or deposition of sandy material, are not only a consequence of the El Niño event, but of the history of occupation and conversion of marginal

No.	Authors	Year	Study Title	Study Location	Target food system	Backbone ecosystem services (ES)	Natural hazards	Consequences to food systems	Resilience Interventions based on ES	Outcomes
										lands, and of inequities in capacities and access to resources for implementing improved soil management and diversification.
				Central and southern Malawi	Small-scale food production, primarily of rein-fed maize agriculture. Systems of maize production range from	Mineral substances (soil) for agricultural purposes.	Droughts and floods.	The inter-seasonal distribution of rainfall had severe impacts on crop production. Low crop yields lead to food	Diversification of production system. Conservation agriculture to improve soil health.	Poor conservation agricultural performance during short trial periods can create

No.	Authors	Year	Study Title	Study Location	Target food system	Backbone ecosystem services (ES)	Natural hazards	Consequences to food systems	Resilience Interventions based on ES	Outcomes
					conventional mono-cultural and tillage-based system to 'conservation agriculture' practice, in which minimum tillage is combined with inter-cropping of legumes and the maintenance of permanent organic soil.			shortage, affect livelihoods and the abilities of farmers to maintain soil conservation practices.		negative perceptions and undermine the value of long-term investment in soil health.
				Mount Wilhelm, Papua New Guinea	Small-scale subsistence agricultur	Cultivated terrestrial plants, soil.	Extreme high temperatures at lower	The El Niño event impacted crops both	Planting closer to water source, in shaded	Return to more favorable agricultural

No.	Authors	Year	Study Title	Study Location	Target food system	Backbone ecosystem services (ES)	Natural hazards	Consequences to food systems	Resilience Interventions based on ES	Outcomes
					e in the forest, garden plots to grow food crops, including staples (sweet potato, banana, and taro). Cash crop production of betel nut and cacao in the lowlands, coffee in the highlands.		elevations (200 m and 700m), coinciding with bush fires and severe drought; at mid-elevation (1200m and 2200 m), reductions in dry season rainfall and increases in temperature were less severe, due to the mediation of cloud effects, and	directly through drought and frost, and indirectly, through changes in ecosystem services and disservices, including pest pressure and predation of pests.	areas, and closer to forests (a strategy which is thought to decrease pest pressure). Awareness of coping strategies existed, such as planting resistant crop varieties or those that can be stored, and employing soil management practice (e.g. mulching). Village and clan-level safety nets	conditions was recorded, few of these coping strategies were maintained as regular agricultural or social practices.

No.	Authors	Year	Study Title	Study Location	Target food system	Backbone ecosystem services (ES)	Natural hazards	Consequences to food systems	Resilience Interventions based on ES	Outcomes
							intermittent frosts occurred at particularly high elevations.		(food and resource sharing, borrowing), along with buying food externally at markets, possible because the drier EL Nino conditions roads remained accessible.	

No.	Authors	Year	Study Title	Study Location	Target food system	Backbone ecosystem services (ES)	Natural hazards	Consequences to food systems	Resilience Interventions based on ES	Outcomes
				Central Region, Ghana	Cocoa growing region, with its forest agricultural landscape with few patches of remnant tree outside the designed forest area.	Cultivated terrestrial plants, soil	Complicated pattern of rainfall across the bi-modal wet season.	Up to 30% mortality of productive cocoa trees, correlating with max. temperature anomalies 10-150C above average. Fungal attacks (black pod) were lower, but insect attacks (capsids) showed complicated pattern.	Farmers noted that fires contributed to a diversification of crop portfolios. Burnt cocoa was replaced with oil palm. Shifting of cultivation of food crops to wetland areas.	Most cocoa plots remained intact, long-term hydrological impact of farming on wetlands and potentially maladaptiveness of this short-term response leading to longer term decrease in resilience.

No.	Authors	Year	Study Title	Study Location	Target food system	Backbone ecosystem services (ES)	Natural hazards	Consequences to food systems	Resilience Interventions based on ES	Outcomes
4	Mubaya CP, Ndebele-Murisa MR. (Mubaya & Ndebele-Murisa, 2020)	2020	The role of ecosystem services in offsetting effects of climate change in sustainable food systems in the Zambezi Basin, Southern Africa	Omay Communal lands in Zimbabwe, total area of 2870 km^2.	Semi-arid lands with low agricultural potential. Small-scale horticulture and crop production	Forest (fruits, poles, firewood/ wood), wild animals (bush meat), land (vegetable and other crop production, rivers (fish, water).	Extreme weather events: flooding, droughts, frost, low rainfall/ high temperature (1968-to date)	Food insecurity, drying up of trees, low fruit production, fish death, decreased water for wildlife and humans, reduced access to wild foods, reduced production, increased poaching, reduced prey for wild animals.	People cope to drought by resorting to consumption of wild fruits (musiga, masarahwa, mupandakata, etc.) and eating vegetables without ant starch.	Locals suggested as actions: Promoting water harvesting techniques and irrigation schemes. Increasing community management of natural resources. There are certain policies are already in place: preventing poisonin

No.	Authors	Year	Study Title	Study Location	Target food system	Backbone ecosystem services (ES)	Natural hazards	Consequences to food systems	Resilience Interventions based on ES	Outcomes
										g of fish, veldt fires, cutting down of trees, cultivation of prohibited lands, self-allocation of settlement.

No.	Authors	Year	Study Title	Study Location	Target food system	Backbone ecosystem services (ES)	Natural hazards	Consequences to food systems	Resilience Interventions based on ES	Outcomes
5.	Meuwissen MPM, Feindt PH, Spiegel A, Termeer CJAM, Mathijs E, Mey Yd, et al. (Meuwissen et al., 2019)	2019	A framework to assess the resilience of farming systems	Netherlands, Veenkolonien regional arable farming system with family farms, including the local potato processing cooperative.	The main products are starch potatoes, sugar beet and winter wheat.	The peat soils dominant in the region shape the arable farmers' planting plans. Farmers assessed the performance of public goods such as soil quality and biodiversity as relatively poor, implying that the system might be robust but	Weather shocks e.g., excessive precipitation leads to landslides.	Poor economic performance per ha, poor performance of public goods.	Relatively high capacity to adapt; many innovations implemented (soil structure, renewable energy).	

No.	Authors	Year	Study Title	Study Location	Target food system	Backbone ecosystem services (ES)	Natural hazards	Consequences to food systems	Resilience Interventions based on ES	Outcomes
						does not provide the right functions.				

No.	Authors	Year	Study Title	Study Location	Target food system	Backbone ecosystem services (ES)	Natural hazards	Consequences to food systems	Resilience Interventions based on ES	Outcomes
6	Sen LTH, Bond J, Winkels A, Linh NHK, Dung NT. (Sen et al., 2021)	2020	Climate change resilience and adaption of ethnic minority communities in the upland area in Thừa Thiên-Huế province, Vietnam	Poor and small-holder farmers form the upland areas of Thừa Thiên-Huế province, Vietnam. The poverty rate is 25% in 2017.	Agricultural production and collection of forest products.	Soil, timber and non-timber collection from natural forests (rattan, herbs, medicinal plants, honey). Forests provide soil conservation and stabilizing stream flows and water runoff.	Extreme weather events, particularly drought and irregular rainfall distribution.	Crop loss, limited arable lands, land degradation due to landslide, soil erosion, expensive production inputs.	Local communities set up various groups to help each other, such as goat keeping groups; acacia groups; non-timber collection groups. Adjustment of the seasonal calendar was the most popular measure adopted by households in the studied area. Mixed cropping; using	The forest dependency group had a relatively higher climate resilience index score, particularly in relation to income and adaptive capacity. Agricultural households had lower income, lower food availability. Most

No.	Authors	Year	Study Title	Study Location	Target food system	Backbone ecosystem services (ES)	Natural hazards	Consequences to food systems	Resilience Interventions based on ES	Outcomes
									tolerant varieties/breeds and storing feed for livestock during winter. Various soil conservation techniques were adopted to protect land from soil and enhance soil fertility: making different types of terraces; planting nitrogen-fixing crops; applying organic	of adaptation measures are indigenous and of low financial requirement.

No.	Authors	Year	Study Title	Study Location	Target food system	Backbone ecosystem services (ES)	Natural hazards	Consequences to food systems	Resilience Interventions based on ES	Outcomes
									fertilizers. Tree planting was an adaptation measure normally employed by households who kept livestock or had fishponds. Planting trees around the garden or the fishpond reduces heat stress and water evaporation	

No.	Authors	Year	Study Title	Study Location	Target food system	Backbone ecosystem services (ES)	Natural hazards	Consequences to food systems	Resilience Interventions based on ES	Outcomes
7	Homann-Kee Tui S, Valdivia RO, Descheemaeker K, Senda T, Masikati P, Makumbe MT, et al. (Homann-Kee Tui et al., 2020)	2020	Crop-livestock integration to enhance ecosystem services in sustainable food systems.	Semiarid Nkayi district, Central Zimbabwe	Rainfed mixed crop-livestock farming. Mainly under maize, with smaller portions of sorghum, groundnuts, and cowpeas as staple crops, combined with the use of communal rangelands, fallow land, and crop residues for livestock productio	Soils, biomass to feed livestock.	Erratic rainfall with a drought year frequency of two in every 5 years.	Low productivity because of a combination of factors: unfavorable climatic conditions, poor and depleted soils, environmental degradation, and low level of capital endowment, leading to limited uptake of improved technologies as well as poor market access and	Crop-livestock system integration enhancing ecosystem services: provisioning (increased cereal, legume and milk yields); supporting (manure replacing inorganic fertilizer, locally produced feed for livestock); regulating (soil improvement, grazing distances reduced, pests	Knowledge and networks improved; farm net returns increased, poverty reduced.

No.	Authors	Year	Study Title	Study Location	Target food system	Backbone ecosystem services (ES)	Natural hazards	Consequences to food systems	Resilience Interventions based on ES	Outcomes
					n (cattle, donkeys, goats).			adverse policies.	prevented, methane emission reduced).	

No.	Authors	Year	Study Title	Study Location	Target food system	Backbone ecosystem services (ES)	Natural hazards	Consequences to food systems	Resilience Interventions based on ES	Outcomes
8.	Tran N, Cao QL, Shikuku KM, Phan TP, Banks LK.(Tran et al., 2020)	2020	Profitability and perceived resilience benefits of integrated shrimp-tilapia-seaweed aquaculture in North Central Coast, Vietnam	Hoang Phong commune in Thanh Hoa province within the North Central Coast region of Vietnam.	A typical production system in-volves use of conventional aquaculture practices where there is monoculture of brackish water shrimps (mainly tiger shrimp) or polyculture shrimp with mud-crab in brackish water ponds. The average pond area	Cultivated aquaculture grown for nutritional purposes.	Erratic rainfall combined with increased variability in day and night temperatures, changing in patterns of the 'Tieu Man' flooding.	Increased mortality rates of shrimps and mud-crabs, resulting in a greater risk of aquaculture crop failure.	The shrimp-tilapia-seaweed polyculture intervention was introduced to the existing system.	Integrated shrimp-tilapia-seaweed aquaculture leads to cost-saving for pellet feed, chemicals, and costs of pond preparation. This is explained by ecosystem services generated by tilapia. 96% of project households

No.	Authors	Year	Study Title	Study Location	Target food system	Backbone ecosystem services (ES)	Natural hazards	Consequences to food systems	Resilience Interventions based on ES	Outcomes
					is 2.4 ha per household. Besides aquaculture, food system includes agriculture (rice and vegetables) and livestock with small areas of land.					indicated that due to integrated aquaculture (IA) increased their capacity to assess food because stocking tilapia provided them fish for food and additional income. 92% of surveyed households reported that IA had helped to diversify

No.	Authors	Year	Study Title	Study Location	Target food system	Backbone ecosystem services (ES)	Natural hazards	Consequences to food systems	Resilience Interventions based on ES	Outcomes
										production, increase farm profitability and decrease climatic risks.

No.	Authors	Year	Study Title	Study Location	Target food system	Backbone ecosystem services (ES)	Natural hazards	Consequences to food systems	Resilience Interventions based on ES	Outcomes
9.	Röös E, Bajzelj B, Weil C, Andersson E, Bossio D, Gordon LJ. (Röös et al., 2021)	2021	Moving beyond organic – A food system approach to assessing sustainable and resilient farming	The farm is located in the forest district of central Sweden.	Crops (wheat, oats, rye, gray peas, common beans, vegetables, landrace cereals, legumes, buckwheat, lamb, landrace beef cattle and pigs, a small flock of laying hens), flowering herbs.	Cultivated terrestrial plants for nutrition, materials; reared animals.	The drought in 2018 (driest summer since 1850),	The rye grown on the farm was a landrace crop, less sensitive to drought. A pest event (birds) decimated legume (pea) yields in 2019. Meat production appeared resilient, however, outbreaks of livestock diseases such as foot-and-mouth disease	The farmers substantially increased the overall diversity of agricultural products (from 5 main products in 2015 to 12 in 2019) and other services e.g. plants flower strips to enhance bee pollination, and also the actors involved (from 3 types of buyers to 6).	The case study farm strongly increased its contribution to global food security between 2015 and 2019 from 0.9 to 1.5 persons fed per ha. The farm is still well below the Swedish average of 4.8 persons fed per ha,

No.	Authors	Year	Study Title	Study Location	Target food system	Backbone ecosystem services (ES)	Natural hazards	Consequences to food systems	Resilience Interventions based on ES	Outcomes
								can lead to the loss of entire herds. Income per ha increased slightly for meat, as a result of increased direct sales.	The farm increased its proportion of crops for direct human consumption, rather than animal feed; the amount of energy produced for human consumption per ha increased by 79%, the amount of total protein by 64%, and the amount of complete protein by 95%. The farmer also used some of the	explained by its location in the forest district and organic production. As to climate impact per kg product, for the total amount of food leaving the farm, emissions per kcal reduced substantially (to 1.2 g CO_2 per kcal) as

No.	Authors	Year	Study Title	Study Location	Target food system	Backbone ecosystem services (ES)	Natural hazards	Consequences to food systems	Resilience Interventions based on ES	Outcomes
									crop residues from the failed crops caused by drought in 2018 and pests (legumes) in 2019 as animal feed.	more crops for direct human consumption were added.

No.	Authors	Year	Study Title	Study Location	Target food system	Backbone ecosystem services (ES)	Natural hazards	Consequences to food systems	Resilience Interventions based on ES	Outcomes
10.	D. Campbell (Campbell, 2021)	2021	Environmental change and the livelihood resilience of coffee farmers in Jamaica: A case study of the Cedar Valley farming region	Cedar Valley region, St. Thomas, Jamaica.	Small-scale farmers cultivated a variety of crops: banana, sugar cane, cocoa, citrus, pimento while the latter is comprised of fruits, vegetables, legumes, condiments and roots and tubers. The main cash crop - coffee. Livestock ownership.	Cultivated terrestrial plants; arable land; water resources; regulation of baseline flows and extreme events.	Droughts, storms, and hurricanes.	Poor yield resulting in low production, pest and diseases e.g. coffee leaf rust was the main plant disease.	The cultivation of cash crops (vegetable and fruits) alongside coffee is one strategy that farmers use to challenge the uncertainties associated with coffee production.	Access to good quality farmlands is a concern that farmers see as a potential barrier to future adaptation strategies.

No.	Authors	Year	Study Title	Study Location	Target food system	Backbone ecosystem services (ES)	Natural hazards	Consequences to food systems	Resilience Interventions based on ES	Outcomes
11	Jackson G, McNamara KE, Witt B. (G. Jackson et al., 2020)	2020	"System of hunger": Understanding causal disaster vulnerability of indigenous food systems	The Bedamuni (volcanic foothills) in the Strickland-Bosavi region of Western Province, Papua New Guinea	Cultivation of crops: plantain, yams, taro, sugar cane, various greens (apika, pitpit), and the wild foraging and transplanting /cultivation of sago palms. Seasonal availability of nuts, fruits, greens, and the hunting and trapping of animals remain	Cultivated and wild terrestrial plants and animals; arable land; water resources; regulation of baseline flows and extreme events.	El Nino's (1971/2, 1997/8, 2015/16) cause droughts and fires. Earthquake (1950s, 2018). Floods (many years). Pest and disease outbreaks (pre-1997, post-1997). Strong winds.	Loss of crops, severe reductions in water availability (quality and quantity), loss of gardens and sago swamps. Fish, crocodile, and crustacean populations impacted leading to problems with protein availability. Taro beetles, banana leaf rust,	Transplanting rhizomes from existing gardens to creeks, rivers, swamps, or caves during drought, or through the use of famine foods (e.g., bush yams, black palm shoots, insects, "poisonous nuts", and "soft rocks") and the temporary migration of some villagers to dense	Although significant changes are needed in the food system, they must be built on the strengths that already exist, moving from incremental to transformational in nature.

No.	Authors	Year	Study Title	Study Location	Target food system	Backbone ecosystem services (ES)	Natural hazards	Consequences to food systems	Resilience Interventions based on ES	Outcomes
					vital parts the food system.			earth worms, caterpillars, rats, pigs, deer increasingly impacted food availability.	forests less affected by drought to collect bush food. Application of composting and techniques for new crops and cultivars, and animals husbandry.	

No.	Authors	Year	Study Title	Study Location	Target food system	Backbone ecosystem services (ES)	Natural hazards	Consequences to food systems	Resilience Interventions based on ES	Outcomes
12.	Mavhura E. (Mavhura, 2017)	2017	Applying a systems-thinking approach to community resilience analysis using rural livelihoods: The case of Muzarabani district, Zimbabwe.	Muzarabani is a semi-arid district , Zimbabwe	Smallholder farmers engaged in rainfed farming. Crops grown include maize, small grains, cotton, and tobacco. Livestock (cattle, ship, goats) rearing is also practicing at subsistence level.	Land, water and woodlands are the natural capital from which resources flow and services are derived. Dry forests support wildlife, livestock and regulate other services. Rich alluvial soils along flood plains have attracted smallhold	Floods (2008, the highest magnitude floods between 2000 and 2015); droughts.	Destruction of crops. When floods or droughts occur, Muzarabani is usually flooded by informal traders and dealers from urban areas selling and exchanging food with livestock. This raises the household food expenditure because the small-	Community has adapted in three ways: mudzedze farming practice, dual cropping systems and traditional flood proofing structures. Plant of cash crops (cotton and tobacco); Cotton is preferred because it is drought tolerant while tobacco has a ready market within and	The quest for tobacco production is however, inadvertently exposing the smallholder farmers to river channel shifts. This is because the poor smallholder farmers use wood fuel to cure tobacco. This increases deforestation,

No.	Authors	Year	Study Title	Study Location	Target food system	Backbone ecosystem services (ES)	Natural hazards	Consequences to food systems	Resilience Interventions based on ES	Outcomes
						er farmers. The river network regulates river flooding.		holder farmers have to sell their assets at relatively low prices whilst buying the food at high prices. The high food prices wipe out the cash remittances and indirectly exacerbate household poverty. A tipping point is reached when all the household	outside the district. The smallholder farmers increasingly relying on wild fruits and dried vegetable leaves. Some farmers use destocking, it saves pastures form being over-grazed and enhances animal health. Livestock is also sold to purchase food and farming inputs.	erosion and siltation, that exacerbate river channel shifts.

No.	Authors	Year	Study Title	Study Location	Target food system	Backbone ecosystem services (ES)	Natural hazards	Consequences to food systems	Resilience Interventions based on ES	Outcomes
								assets are depleted.	Farmers use ethno-based early warning systems (appearance of stock birds *Ciconia ciconia*, the abundance of wild fruits *hwakwa* etc.) sometimes accompanied by early temporal migrations to uplands.	

No.	Authors	Year	Study Title	Study Location	Target food system	Backbone ecosystem services (ES)	Natural hazards	Consequences to food systems	Resilience Interventions based on ES	Outcomes
13	Cauchi JP, Moncada S, Bambrick H, Correa-Velez I. (Cauchi, Moncada, Bambrick, & Correa-Velez, 2021)	2021	Coping with environmental hazards and shocks in Kiribati: Experiences of climate change by atoll communities in the Equatorial Pacific.	Six communities in 4 islands in The Republic of Kiribati in the Equatorial Pacific.	Communities heavily reliant on subsistence living, gardening or farming are given great priority.	Trees are highly valued as a food supply and a source of copra (dried coconut kernels), sold for cash. Food gardens and fishing grounds.	Perigean 'King' Tides. Storm surges. Rainwater flooding. Strong winds.	Food tree/crop destruction; decreasing soil fertility. Damage to crops.	Current response: Letting the plants die; trying to use more resilient crops (breadfruit vs. papaya). Waiting for heavy rain to clear soil salinity, increase fertility through composting. Planting mangroves to protect against strong waves; eating imported food.	Alternative response: Planting salt-tolerant crops; sea defenses (mangroves, natural defenses); adding water to soil; protecting soil through raised beds. Using sustainable methods to encourage land growth

No.	Authors	Year	Study Title	Study Location	Target food system	Backbone ecosystem services (ES)	Natural hazards	Consequences to food systems	Resilience Interventions based on ES	Outcomes
									Digging channels to drain water. Planting food trees away from where they fell; building moveable fences; chopping the trees.	(e.g. Te buibui). Using pumps, raised garden plots; proper food storage infrastructure; Maintenance of fences; replanting trees; better protection; maintenance of fences.

No.	Authors	Year	Study Title	Study Location	Target food system	Backbone ecosystem services (ES)	Natural hazards	Consequences to food systems	Resilience Interventions based on ES	Outcomes
14	Rammig A, Bahn M, Vera C, Knoke T, Paul C, Vollan B, et al. (Rammig et al., 2020)	2020	Adaptive capacity of coupled social-ecological systems to absorb climate extremes.	Coastal regions of Ecuador, the Andean region.	Small-scale agricultural production.	Fertile soils, forest	El Nino event (2012) flooding, drought.	Crop yields were completely lost on 50% of the agricultural area in the coastal regions. Another 21% of the area was affected by the flooding.	Existing socio-economic system may buffer effects of climate extremes through land-use allocation and land-use diversification until a certain point.	Measures to increase the adaptive capacity may be to increase economically attractive forestry and agroforestry practices and diversified land-use systems, which offer the potential to diversify income sources as well

No.	Authors	Year	Study Title	Study Location	Target food system	Backbone ecosystem services (ES)	Natural hazards	Conseque nces to food systems	Resilience Interventio ns based on ES	Outcome s
										as ecologica l functions .

No.	Authors	Year	Study Title	Study Location	Target food system	Backbone ecosystem services (ES)	Natural hazards	Consequences to food systems	Resilience Interventions based on ES	Outcomes
15	Prado DS, Seixas CS, Berkes F. (Prado et al., 2015)	2015	Looking back and looking forward: Exploring livelihood change and resilience building in a Brazilian coastal community.	The Aventureiro village is located at Ilha Grande, an island in the municipality of Angra dos Reis, Rio de Janeiro State, southeastern coast of Brazil.	Small-scale fisheries, agriculture, manioc flour milling, home garden production, marine invertebrate gathering.	Community is surrounded by a diverse and well-preserved environment (sand beaches, lagoons, mangroves, forest, rocky shores, sea).	Sea and wind conditions have been the major environmental source of vulnerability in Aventureiro. The establishment of protected area (2010-2014).	Prolonged rough seas make the maritime access impossible with consequences for purchasing most food items on the mainland. It also affects local small-scale fishing, and in face of food shortage, people resort to food loans and sharing networks.	Resilience strategies include tourism-related activities (i.e. camping, boat trips and small restaurants), home garden cultivation and agriculture, non-timber forest products (NTFP) extraction, small-scale/artisanal fishing, marine invertebrates gathering, and chicken	The government's conservation policies were found to be an important driver of change at the local level. The protected areas are main source of disturbance for the households, in terms of land rights, management practices and local

No.	Authors	Year	Study Title	Study Location	Target food system	Backbone ecosystem services (ES)	Natural hazards	Consequences to food systems	Resilience Interventions based on ES	Outcomes
									and other animal raising. Except for tourism, the other activities consisting of livelihood strategies are mostly for subsistence and are therefore an important component of food security.	resource use. The people of Aventureiro have been coping with the "illegality" of their livelihoods, and with various prohibitions and conflicts.

No.	Authors	Year	Study Title	Study Location	Target food system	Backbone ecosystem services (ES)	Natural hazards	Consequences to food systems	Resilience Interventions based on ES	Outcomes
16	Umamaheswari T, Sugumar G, Krishnan P, Ananthan PS, Anand A, Jeevamani JJJ, et al. (Umamaheswari et al., 2021)	2021	Vulnerability assessment of coastal fishing communities for building resilience and adaptation: Evidences from Tamil Nadu, India.	Coastal villages of Thoothukud, a coastal district of Tamil Nadu in India.	Natural-resource based livelihood system, marine fishing – dominating.	Coastal and aquatic biomass used for nutrition.	Climate change in different dimensions. The district is highly vulnerable to cyclones and monsoon causing heavy damages to the coastal areas almost every year in addition to seasonal fluctuations in terms of temperature, relative humidity	Disappearance of existing major fisheries, reduction in fish catch, drastic change in the distribution of fish species, migration of fishes.	Possible interventions: Intensify the access to selective fishing methods, and aquaculture practices; reduction of fishing pressure; technology transfer; Create awareness on conservation and sustainable exploitation of fishery/local resources through co-management;	Enhanced livelihood security and resilience of fisheries to climate vagaries.

No.	Authors	Year	Study Title	Study Location	Target food system	Backbone ecosystem services (ES)	Natural hazards	Consequences to food systems	Resilience Interventions based on ES	Outcomes
							and wind speed.		Encourage fishery diversification/livestock and non-fishery enterprises.	

No.	Authors	Year	Study Title	Study Location	Target food system	Backbone ecosystem services (ES)	Natural hazards	Consequences to food systems	Resilience Interventions based on ES	Outcomes
17	Kaplan-Hallam M, Bennett NJ, Satterfield T. (Kaplan-Hallam, Bennett, & Satterfield, 2017)	2017	Catching sea cucumber fever in coastal communities: Conceptualizing the impacts of shocks versus trends on social-ecological systems.	Rio Lagartos, a fishing community on Mexico's Yucatan Peninsula.	Small-scale commercial fishing with octopus, spiny lobster, red grouper, snapper, and sea cucumber.	Aquatic biomass.	Climate change	Gradually declining fish catches.	Increasing fishing effort, shrimping in the estuary, diversifying livelihoods, cultivating a local eco-tourism industry, new income opportunities within the sea cucumber fishery.	The recent emergence of commercial sea cucumber fishing on the Yucatán Peninsula has driven fast and dramatic changes in the community of Río Lagartos. It has triggered a gold-rush style influx of new actors, new livelihoo

No.	Authors	Year	Study Title	Study Location	Target food system	Backbone ecosystem services (ES)	Natural hazards	Consequences to food systems	Resilience Interventions based on ES	Outcomes
										d opportunities (and risks), creating new pressures on local environmental resources.

No.	Authors	Year	Study Title	Study Location	Target food system	Backbone ecosystem services (ES)	Natural hazards	Consequences to food systems	Resilience Interventions based on ES	Outcomes
18	Jezeer RE, Verweij PA, Boot RGA, Junginger M, Santos MJ.(Jezeer, Verweij, Boot, Junginger, & Santos, 2019)	2019	Influence of livelihood assets, experienced shocks and perceived risks on smallholder coffee farming practices in Peru.	San Martin, Peru. The average farm size is 2.75 ha.	Smallholder coffee farmers.	Cultivated trees, soil. Shade tree species richness and density.	Climate change – higher maximum temperatures and rainfall variability. Increased pest and disease incidence.	The greatest concern of farmers was related to the fluctuating coffee prices rather than extreme climate or pest and disease events.	These pressures lead to opposing strategies: climate change perception motivated farmers to increase shade levels, while pressure from pests and diseases led to a reduction in shade and increasing in inputs. There are indirect benefits of shade trees such as buffering	Adoption of farming practices was more strongly influenced by livelihood assets than perception of risks.

No.	Authors	Year	Study Title	Study Location	Target food system	Backbone ecosystem services (ES)	Natural hazards	Consequences to food systems	Resilience Interventions based on ES	Outcomes
									climate change, soil erosion control, enhanced soil fertility, and improved bean quality.	

No.	Authors	Year	Study Title	Study Location	Target food system	Backbone ecosystem services (ES)	Natural hazards	Consequences to food systems	Resilience Interventions based on ES	Outcomes
19	Williams PA, Crespo O, Abu M. (Williams, Crespo, & Abu, 2019)	2019	Adapting to changing climate through improving adaptive capacity at the local level – The case of smallholder horticultural producers in Ghana.	Keta is a coastal area within the coastal savannah agro-ecological zone; Nsawam is inland within the forest deciduous agro-ecological zone Ghana.	Smallholder farmers growing horticultural crops: okro, tomato, pepper, onions, spring onions, carrots, shallots, cabbage, pineapple and watermelon.	Cultivated terrestrial plants and animals; arable land; water resources.	Keta is particularly sensitive to changing climate associated with temperature and precipitation. Nsawam has been experiencing decreasing precipitation pattern with increasing temperature.	Changing climate mostly affects the quality of horticultural farmer's produce (dehydration, leaf scorches, increase in pest and diseases, variation in fruit maturity and abnormal fruit set), outputs, yield losses after harvesting and overall net revenue.	In Keta the first five most important adaptation practices at the local level included fertilization, supplementary irrigation, mixed cropping, crop rotation, and intercropping. For Nsawam's practices also showed fertilization as the first highest ranked and important	Practicing fertilization (both organic and inorganic), which enhances nutrient availability and improves soil fertility was the most important to horticultural households. The top crop management practices are crop rotation,

No.	Authors	Year	Study Title	Study Location	Target food system	Backbone ecosystem services (ES)	Natural hazards	Consequences to food systems	Resilience Interventions based on ES	Outcomes
									adaptation practices, followed by crop rotation, mixed cropping, supplementary irrigation and intercropping. Other adaptation strategies include: decrease/increase farm size, mixed farming, weather forecast, use improved varieties, adjustment of planting calendar, crop	mixed cropping and intercropping identified are mainly to improve soil and water use efficiency, enhance nutrient uptake and buffer against losses in an unfavorable season. horticultural Smallholders' were financially

No.	Authors	Year	Study Title	Study Location	Target food system	Backbone ecosystem services (ES)	Natural hazards	Consequences to food systems	Resilience Interventions based on ES	Outcomes
									diversification, make ridges, wind break structures, shifting cultivation, mulching, build traditional dams, drainage construction, shift into animal production, integrated pest management, livelihood diversification, organic farming, crop insurance.	constrained and limited in their capacity to practice the identified adaptation strategies to its full potential.

No.	Authors	Year	Study Title	Study Location	Target food system	Backbone ecosystem services (ES)	Natural hazards	Consequences to food systems	Resilience Interventions based on ES	Outcomes
20	Marie M, Yirga F, Haile M, Tquabo F. (Marie, Yirga, Haile, & Tquabo, 2020)	2020	Farmers' choices and factors affecting adoption of climate change adaptation strategies: evidence from northwestern Ethiopia.	Gondar Zuria District characterized by a semi-arid climate, Central Gondar Zone, Amhara Regional State, northwest Ethiopia.	Mixed farming is predominant (i.e. crop production and livestock rearing (90%). Major crops include wheat, sorghum, pea, teff, maize, and others. The livestock population in the district is equivalent to 207,000 tropical livestock units.	Cultivated terrestrial plants and animals; arable land; water resources.	Climate variability and extreme weather events.	Crop failure, severe soil erosion and shortages of water.	Farmers have implemented mixed farming, mixed cropping, early and late planting (changing sowing period), use of drought-resistant crop varieties, application of soil and water conservation techniques, shifting to non-farm income activities and use of irrigation.	Future resilience strategies should focus on improving climate change information access, improving market access and enhancing research on the use of rainwater harvesting technology.

No.	Authors	Year	Study Title	Study Location	Target food system	Backbone ecosystem services (ES)	Natural hazards	Consequences to food systems	Resilience Interventions based on ES	Outcomes
21	Atara A, Tolossa D, Denu B.(Atara, Tolossa, & Denu, 2020)	2020	Analysis of rural households' resilience to food insecurity: Does livelihood systems/choice/ matter? The case of Boricha woreda of sidama zone in southern Ethiopia.	Boricha woreda, which is located in the western part of the Sidama Zone in Southern Ethiopia. Boricha woreda covers 588.1 sq. km.	Both enset and maize are dominant food crops. Livestock, khat, and coffee are also part of the household's economy.	People largely depend on man-made ponds for both humans and livestock.	Erratic rainfall patterns. Drought for a period 2002-2005.	Reliance on rain-fed farming coupled with rainfall variability leads to harvest loss. Households lost their crops due to drought and experienced a chronic food shortage.	Agro-pastoralists of the Borich woreda (district) can be regarded as half agriculturalists and half pastoralists. They plant staple food crops such as maize and even cash crops such as khat. It is one with a relatively less land fragmentation and consists of households keeping large	Looking for a way of addressing population pressure on land or improving agricultural asset possessions of individual households can be good intervention priority for coffee and maize livelihood systems.

No.	Authors	Year	Study Title	Study Location	Target food system	Backbone ecosystem services (ES)	Natural hazards	Conseque nces to food systems	Resilience Interventio ns based on ES	Outcome s
									number of livestock, specially cattle.	

No.	Authors	Year	Study Title	Study Location	Target food system	Backbone ecosystem services (ES)	Natural hazards	Consequences to food systems	Resilience Interventions based on ES	Outcomes
22	Hernandez Y, Guimarães Pereira Â, Barbosa P. (Hernandez, Guimaraes Pereira, & Barbosa, 2018)	2018	Resilient futures of a small island: A participatory approach in Tenerife (Canary Islands) to address climate change.	The Tenerife Island (Canary Islands)	—	Soil, water, cultivated plants	Extreme weather events – heatwaves, dust events.	The level of food self-sufficiency is considered low and decreasing	The need to increase local agricultural production based on sustainable ways, especially to improve soil quality. To adjust water demand to the biophysical capacities of natural water in Tenerife.	

No.	Authors	Year	Study Title	Study Location	Target food system	Backbone ecosystem services (ES)	Natural hazards	Consequences to food systems	Resilience Interventions based on ES	Outcomes
23	Paumgarten F, Locatelli B, Witkowski ETF, Vogel C. (Paumgarten, Locatelli, Witkowski, & Vogel, 2020)	2020	Prepare for the unanticipated: Portfolios of coping strategies of rural households facing diverse shocks.	The villages of Bennde Mutale and Vondo in the Vhembe District Municipality of South Africa's Limpopo Province, one of the country's poorest provinces.	Small-scale farming.	Cultivated and wild terrestrial plants and animals; arable land; water resources.	Natural hazards, including floods, droughts.	Crop and livestock losses; crop and livestock pests/diseases; loss of agricultural/grazing land.	41% of households relied on the safety-net function of natural products (e.g. non-timber forest products from non-cultivated ecosystems).	Natural products were not often used to cope with natural hazards, suggesting limitations to their safety-net function for covariate, climate-related shocks. By relying on natural products to buffer against shocks,

No.	Authors	Year	Study Title	Study Location	Target food system	Backbone ecosystem services (ES)	Natural hazards	Consequences to food systems	Resilience Interventions based on ES	Outcomes
										households may be better able to retain their savings for natural hazards.

No.	Authors	Year	Study Title	Study Location	Target food system	Backbone ecosystem services (ES)	Natural hazards	Consequences to food systems	Resilience Interventions based on ES	Outcomes
24	Joffre OM, Bosma RH, Bregt AK, van Zwieten PAM, Bush SR, Verreth JAJ. (Joffre et al., 2015)	2015	What drives the adoption of integrated shrimp mangrove aquaculture in Vietnam?	The Mekong Delta, Vietnam.	Smallholder shrimp aquaculture.	Mangrove forests, coastal ecosystems.	Nature-induced changes in coastal landscape and mangrove ecosystems (changes in hydrological system causing coastal erosion or accretion and sea level rise).	Decreasing of productivity of shrimp aquaculture.	Integrated mangrove-shrimp production systems can contribute to rebuilding resilience in the Mekong Delta.	Experts considered the ecosystem function of the mangrove an enabling driver pushing farmers to plant mangrove in order to improve the pond's water quality and limit disease outbreaks.

No.	Authors	Year	Study Title	Study Location	Target food system	Backbone ecosystem services (ES)	Natural hazards	Consequences to food systems	Resilience Interventions based on ES	Outcomes
25	Makwinja et al. (Makwinja et al., 2021)	2021	Lake Malombe fishing communities' livelihood, vulnerability, and adaptation strategies.	Lake Malombe, the Southern part of Lake Malawi.	Fishery. Cichlidae is the most dominant, followed by Claridae, then Cypri-nidae. Bathyclarias, Fossorochromis, Pseudotropheus, Caprichromis, and Brycinus contribute the least.	Inland tropical shallow lake providing aquatic resources.	Climate change: heavy rain, drought, and heavy winds.	Significant mass depletion of fish biomass, leading to limited access to food throughout or part of the year.	When Lake Malombe fishery eventually collapsed, some fishers diversified their livelihood by cultivating the upland areas and steep slopes, some few kilometers away from the shoreline. Others diversified into flood plain cultivation (Dimba), small-scale irrigation along the	Fishers do not cooperate to manage the ecosystem during periods of low catches. Instead, they engage in illegal strategies, which is in the long run, deteriorate the ecosystem. To break the high dependency, it is necessary to promote

No.	Authors	Year	Study Title	Study Location	Target food system	Backbone ecosystem services (ES)	Natural hazards	Consequences to food systems	Resilience Interventions based on ES	Outcomes
									shoreline. Another group took the opportunity to venture into business, lending money to independent fishers with a massive interest of about 30%. Others could join cooperatives that support them during the low catch, while others could run small businesses.	sustainable proactive, adaptive capacity in the fishing communities while acknowledging the link between social and ecological resilience.

No.	Authors	Year	Study Title	Study Location	Target food system	Backbone ecosystem services (ES)	Natural hazards	Consequences to food systems	Resilience Interventions based on ES	Outcomes
26	Asante F, Guodaar L, Arimiyaw S. (Asante, Guodaar, & Arimiyaw, 2021)	2021	Climate change and variability awareness and livelihood adaptive strategies among smallholder farmers in semi-arid northern Ghana.	Vulnerable rural farming communities in the Mamprugu-Moagduri District in the northern semi-arid regions of Ghana, having the poorest agro-ecology in the country.	A total of 121 smallholder farmers depend on rain-fed agriculture. Main crops: maize, groundnuts, rice.	Cultivated terrestrial plants and animals; arable land; water resources.	Climate change: protracted drought, unpredictable rainfall pattern, high temperature, strong winds, and frequent flood events.	Decreased crop output.	Smallholders employ preferred livelihood adaptive strategies involving non-farm diversification (51.2%), crop diversification (75.2%), changes in farm location (76.9%) and agrochemical application (100%) with moderate or high levels of effectiveness across space.	The increasing demand for charcoal and firewood in rural communities due to inadequate finance for efficient energy involved indiscriminate cutting down of trees, which could affect biodiversity. Such livelihood

No.	Authors	Year	Study Title	Study Location	Target food system	Backbone ecosystem services (ES)	Natural hazards	Consequences to food systems	Resilience Interventions based on ES	Outcomes
										strategies, though, could increase vulnerability or reduce smallholders' adaptive capacity, have the potential to improve the livelihood resilience of many rural populations at the household level. There is a need for a location-specific

No.	Authors	Year	Study Title	Study Location	Target food system	Backbone ecosystem services (ES)	Natural hazards	Consequences to food systems	Resilience Interventions based on ES	Outcomes
										climate adaptation policy, using local knowledge.

No.	Authors	Year	Study Title	Study Location	Target food system	Backbone ecosystem services (ES)	Natural hazards	Consequences to food systems	Resilience Interventions based on ES	Outcomes
27	Pei-Shan Sonia Lin (Lin, 2019)	2019	Building resilience through ecosystem restoration and community participation: Post-disaster recovery in coastal island communities.	Koh Klang (Kang Island), a mangrove-luxuriant island on Thailand's Andaman coast. 26 km^2 area.	Small-scale coastal and shallow water fishing and organic rice agriculture.	Mangrove ecosystems providing a variety of ES such as biogeochemical cycling, carbon sequestration, livelihood support, and disaster protection.	Flooding from Indian Ocean tsunami (2004)	Tsunami flooded the island, damaged houses along the shore, and washed away villagers' boats, fish rafts, and farms.	The mangroves increase island resilience in several respects. In addition to providing shelter for the mud crab, one of the most important crabs with a good market price, they serve as a nursery for all aquatic animals. The mangroves protect the canal banks from erosion and help reduce wave	The findings show that after a disaster, natural resources and embedded social norms form the basis for a resilient community. Villagers' dependence on the mangroves resulted in enduring informal social norms

No.	Authors	Year	Study Title	Study Location	Target food system	Backbone ecosystem services (ES)	Natural hazards	Consequences to food systems	Resilience Interventions based on ES	Outcomes
									impacts on the seashore.	for sustainably managing the ecosystem. For example, the villagers understand that they can only fell a few trees for domestic use, such as repairing bungalows, and that they must plant ten new trees when they take one tree.

No.	Authors	Year	Study Title	Study Location	Target food system	Backbone ecosystem services (ES)	Natural hazards	Consequences to food systems	Resilience Interventions based on ES	Outcomes
										Instead of creating rules or setting penalties for illegal cutting, simply increasing the awareness of the importance of mangroves helps people desire to maintain the mangroves.

No.	Authors	Year	Study Title	Study Location	Target food system	Backbone ecosystem services (ES)	Natural hazards	Consequences to food systems	Resilience Interventions based on ES	Outcomes
28	Moreno J, Lara A, Torres M. (Moreno, Lara, & Torres, 2019)	2019	Community resilience in response to the 2010 tsunami in Chile: The survival of small-scale fishing community.	El Morro, Talcahuano region, the south-central coast of Chile.	Small-scale fishing community.	Aquatic biomass.	Earthquake and tsunami (2010)	The totality of houses and fishing boats were swept away. People were starving and they did not have food because the tsunami swept away all their provisions.	Five main themes emerged from the resilience capacities and were identified from the data analysis: (i) local knowledge; (ii) sense of community; (iii) social capital; (iv) cooperation and organization; and (v) trust.	

No.	Authors	Year	Study Title	Study Location	Target food system	Backbone ecosystem services (ES)	Natural hazards	Consequences to food systems	Resilience Interventions based on ES	Outcomes
29	Ha'apio MO, Gonzalez R, Wairiu M. (Ha'apio, Gonzalez, & Wairiu, 2019)	2019	Is there any chance for the poor to cope with extreme environmental events? Two case studies in the Solomon Islands.	Rural community from Ranogha Island in the Western Province of the Solomon Islands; Flood-prone area along the Mataniko river side in peri-urban Honiara.	Farm-house model under semi-subsistence regime i.e. people cropped and fished for their own consumption, and production surpluses were mostly for sharing rather than for trading.	In the riverside location people found enough land to grow their own food, space to build their homes and surrounding land to collect firewood. In the case of the coastal rural community, their lives have traditionally been focused on land and fishing	Tsunami (April 2007); Flash flood (April 2014).	The flood damaged houses, food gardens and sources of livelihood.	Villagers from the peri-urban location depend basically on casual wages and food gardening. Their choices for cropping, fishing or collecting food from the surrounding natural resources are limited. Unlike households in the peri-urban location, households in the rural village live under	In spite of their poverty, households from both locations manage to survive, relying either on home-produced food or food-gathering from the surrounding environment. Even in the peri-urban location, they chose the flood-prone

No.	Authors	Year	Study Title	Study Location	Target food system	Backbone ecosystem services (ES)	Natural hazards	Consequences to food systems	Resilience Interventions based on ES	Outcomes
						grounds from which to make a living.			subsistence conditions, basically relying on what they collect, crop, fish or share; they do not depend largely on cash to survive.	area because of the availability of land for growing their own food. The availability of food collected from the surrounding environment and of land on which to grow their own food is even more important as source of livelihoo

No.	Authors	Year	Study Title	Study Location	Target food system	Backbone ecosystem services (ES)	Natural hazards	Consequences to food systems	Resilience Interventions based on ES	Outcomes
										d than their precarious cash income.

No.	Authors	Year	Study Title	Study Location	Target food system	Backbone ecosystem services (ES)	Natural hazards	Consequences to food systems	Resilience Interventions based on ES	Outcomes
30	Ha'apio MO, Gonzalez R, Wairiu M. (Basupi, Quinn, & Dougill, 2019)	2019	Adaptation strategies to environmental and policy change in semi-arid pastoral landscapes: Evidence from Ngamiland, Botswana.	The southern fringes of the Okavango Delta in Ngamiland District, North-western Botswana.	Pastoral and agro-pastoral communities.	Land use types are affected by environmental factors such as the distribution of surface water and soil quality, regulating the spatial distribution of cattle, wildlife, and dryland and floodplain cultivation.	Droughts	The impacts on pasture regeneration, rainfed arable agriculture and the impact of societal reliance on ecosystem services. Other constraints associated with low rainfall were defined in terms of availability of potable water for livestock, with	Livelihood diversification involves the creation of a portfolio of non-pastoral livelihood activities: livestock diversification (cattle, sheep, goats, donkeys and horses); fishing; flood recession cultivation; fodder accumulation.	Landscape fragmentation and a lack of market access threaten the sustainability of rangelands and challenge the practice of pastoral mobility. While this might be fostering a rise in livelihood diversification through non-

No.	Authors	Year	Study Title	Study Location	Target food system	Backbone ecosystem services (ES)	Natural hazards	Consequences to food systems	Resilience Interventions based on ES	Outcomes
								ephemeral water sources especially congested during dry years, while ground water sources saline and not suitable for livestock.		pastoral activities, some of these strategies might actually undermine the long-term sustainability of pastoralism and complicate responses to natural hazards.

No.	Authors	Year	Study Title	Study Location	Target food system	Backbone ecosystem services (ES)	Natural hazards	Consequences to food systems	Resilience Interventions based on ES	Outcomes
31	Jellason NP, Baines RN, Conway JS, Ogbaga CC. (Jellason, Baines, Conway, & Ogbaga, 2019)	2019	Climate Change Perceptions and Attitudes to Smallholder Adaptation in Northwestern Nigerian Drylands.	Zango and Kofa dryland smallholders in northwest of Nigeria.	In Zango the production of cereal and legume crops: sorghum, pearl millet, cowpea, soybean, groundnuts. In Kofa farmers involved mainly in production of maize, sorghum, millet, cowpea, soybean, groundnuts and some vegetables (onion,	Cultivated terrestrial plants; regulation of soil quality; surface water used for nutrition and irrigation.	Climate change - drought	Poor fertility of most soils; decrease in arable yield; decreasing in crops and livestock.	A few households had adopted irrigation in Kofa, compared to no uptake in Zango. Most respondents in Kofa and all respondents in the Zango adopted agroforestry and improved varieties of crops due to access to improved seeds. Uptake of intercropping was 93% and of crop	Good practices such as mulching for water and fertility management, rehabilitating problems of soil sealing and compaction were poorly adopted. Despite the presence of irrigation facilities in the outskirts of the Zango communi

No.	Authors	Year	Study Title	Study Location	Target food system	Backbone ecosystem services (ES)	Natural hazards	Consequences to food systems	Resilience Interventions based on ES	Outcomes
					tomato, garlic.				rotation was 80% in Zango, compared to 67% and 44% in the Kofa.	ty, the type of agriculture practiced was 100% rainfed; this was also the dominant in Kofa, with 7% applied irrigation. Adaptation practices important for dryland resilience were visibly absent in both communities.

No.	Authors	Year	Study Title	Study Location	Target food system	Backbone ecosystem services (ES)	Natural hazards	Consequences to food systems	Resilience Interventions based on ES	Outcomes
32	Ellen Woodley (Woodley, 2011)	2011	Building Nigeria's Response to Climate Change: Pilot Projects for Community-Based Adaptation in Nigeria.	15 communities in Nigeria, ranging from the Sahel in Yobe state in northeastern Nigeria to the coastal zone in Cross River State in the southeast.	Small-scale farming activities, livestock in Sahel.	Farmland and grazing lands, surface water in Sahel.	Climate change. Aridity and severe weather (drought and sandstorm) in the Sahel.	Invading mobile sand dunes are now encroaching on farmland and grazing lands in Sahel.	Improved varieties of cowpea, sorghum, millet groundnut, maize and rice were introduced. Farmers are also being encouraged to grow resilient wild food tree species of high traditional value both on farm and in home compounds. Sand dune stabilization has been initiated with the planting of	Some projects aim to increase adaptive capacity by establishing alternative livelihood options, while some projects tackle the climate change impacts directly, by introducing drought resistant crop varieties and

No.	Authors	Year	Study Title	Study Location	Target food system	Backbone ecosystem services (ES)	Natural hazards	Consequences to food systems	Resilience Interventions based on ES	Outcomes
									15,000 seedlings of fast growing *Prosopis juliflora* on dunes to provide wood, food and livestock feed.	setting up water supply options. Efforts to increase adaptive capacity must include complimentary efforts to increase awareness within communities of the importance of local ecological functions for sustaining human life and livelihoods.

No.	Authors	Year	Study Title	Study Location	Target food system	Backbone ecosystem services (ES)	Natural hazards	Conseque nces to food systems	Resilience Interventio ns based on ES	Outcome s

No.	Authors	Year	Study Title	Study Location	Target food system	Backbone ecosystem services (ES)	Natural hazards	Consequences to food systems	Resilience Interventions based on ES	Outcomes
	Ellen Woodley (Woodley, 2011) – cont.			Sudan savanna and the Guinea savanna.	Small-scale farming.	Soil, surface water, fuel wood.	Sustained reduction in rainfall.	Declining crop productivity.	Introducing improved varieties of the main crops of sorghum, millet, and cowpea; Application of organic fertilizers.	Yields of all three crops were above average in the 2010, shortening the period of hunger that occurs before harvest each year.

No.	Authors	Year	Study Title	Study Location	Target food system	Backbone ecosystem services (ES)	Natural hazards	Consequences to food systems	Resilience Interventions based on ES	Outcomes
	Ellen Woodley (Woodley, 2011) – cont.			The Guinea savanna.	Small-scale farming.	Water	Increasing in the annual average temperature and erratic rainfalls.	Water scarcity and food insecurity; increased diseases in livestock; lower crop yields.	The dam reservoir was deepened in order to supplement dry season water supply; Improved varieties of maize, cow pea, groundnut and soya bean were introduced; Trees planting; Using soya bean to control the parasitic vine, *Striga asiatica*.	

No.	Authors	Year	Study Title	Study Location	Target food system	Backbone ecosystem services (ES)	Natural hazards	Consequences to food systems	Resilience Interventions based on ES	Outcomes
	Ellen Woodley (Woodley, 2011) – cont.			The rainforest/ coastal ecozones of Cross River State	Fishing supplements agriculture. The main crops: cassava, yam, cocoa yam.	Mangroves, which are important as buffers to the direct impact from storms and associated erosion from severe weather, as well providing habitat for diverse aquatic species.	Climate change impact – sea level rise and possible salt water intrusion onto farm land.	Low fish stocks; reduction in crop yield.	Introducing aquaculture as the adaptation strategy; Introducing alternative livelihood strategies – cassava (gari processing) and snail farming to replace bush meat.	

No.	Authors	Year	Study Title	Study Location	Target food system	Backbone ecosystem services (ES)	Natural hazards	Consequences to food systems	Resilience Interventions based on ES	Outcomes
33	Gordon LJ, Enfors EI. (Gordon & Enfors, 2008)	2008	Land degradation, ecosystem services and resilience of smallholder farmers in Makanya catchment, Tanzania.	Land degradation, ecosystem services and resilience of smallholder farmers in Makanya catchment, Tanzania	Smallholder subsistence farmers, an average 80% of all food eaten is produced within households.	Provisioning ES: food, fuelwood and wild fruit.	Drought. Two consecutive low-rainfall 'masika' rains seasons (2005, 2006).	Dramatic yield losses. Households experienced food shortages, which meant that they had to adopt coping strategies, such as changing their diets, reducing the amount of food per meal and/or reducing the number of meals eaten per day. Cash crops such	Only 20% of household food requirements were met by their own farming systems (including this season's harvests, storage from previous harvests, poultry and livestock. Harvesting wild-growing fruits and vegetables contributed 11%.	A substantial contribution to household nutrition therefore came from the local environment capacity to generate goods that could provide an alternative income when harvests failed. In the study area, 85% of the

No.	Authors	Year	Study Title	Study Location	Target food system	Backbone ecosystem services (ES)	Natural hazards	Consequences to food systems	Resilience Interventions based on ES	Outcomes
								as tomatoes, onions, cabbages and beans, which normally serve as the main income source in the area, were also affected by the lack of rain. Making people increasingly reliant on income sources that were less rainfall-dependent, such as livestock and forest products,		interviewed households earned incomes based on locally generated provisioning ecosystem services such as fibre, wood products, wild fruit, and fodder for free-ranging livestock. On average, more than 40% of the

No.	Authors	Year	Study Title	Study Location	Target food system	Backbone ecosystem services (ES)	Natural hazards	Consequences to food systems	Resilience Interventions based on ES	Outcomes
								although livestock prices dropped dramatically during this time.		total incomes came from these sources, making it the most important income sector in the area. This illustrates the dependence of smallholders on the local ecosystem, despite a growing consensus that income diversification towards

No.	Authors	Year	Study Title	Study Location	Target food system	Backbone ecosystem services (ES)	Natural hazards	Consequences to food systems	Resilience Interventions based on ES	Outcomes
										non-agroecosystem sources is an increasingly important strategy to cope with drought.

No.	Authors	Year	Study Title	Study Location	Target food system	Backbone ecosystem services (ES)	Natural hazards	Consequences to food systems	Resilience Interventions based on ES	Outcomes
34	Quandt A, Neufeldt H, McCabe J. (Quandt, Neufeldt, & McCabe, 2017)	2017	The role of agroforestry in building livelihood resilience to floods and drought in semiarid Kenya.	The communities of Burat and Kinna in Isiolo County, Kenya.	Smallholder farmers.	Tree species. Mango, papaya, banana trees as most important.	Climate change – floods, droughts.	Livestock hunger, disease, death; destruction of crops.	Resilience benefits provided by trees: fruit and food for sale and consumption; construction material; shade for farm; drought resistant trees; firewood; windbreak; cover from rain; climb trees to escape water; prevent soil erosion and reduces water flow; medicine; fodder; compost.	The major livelihood benefits during both floods and drought were shade and fruit for sale and consumption. Agroforestry can provide an alternative source of food and income during floods

No.	Authors	Year	Study Title	Study Location	Target food system	Backbone ecosystem services (ES)	Natural hazards	Consequences to food systems	Resilience Interventions based on ES	Outcomes
										and drought.

No.	Authors	Year	Study Title	Study Location	Target food system	Backbone ecosystem services (ES)	Natural hazards	Consequences to food systems	Resilience Interventions based on ES	Outcomes
35	Saxena A, G¸neralp B, Bailis R, Yohe G, Oliver C. (Saxena, G¸neralp, Bailis, Yohe, & Oliver, 2016)	2016	Evaluating the Resilience of Forest Dependent Communities in Central India by Combining the Sustainable Livelihoods Framework and the Cross Scale Resilience Analysis.	The village panchayat is located about 55 km south of the Bhopal, India.	Significant population within the region lives below poverty line and subsists with a combination of rain-fed agriculture, manual labor, and extraction and utilization of forest-based resources.	Agricultural lands, six open-cast stone quarries; forests. Species like Tendu (*Diasporos melanoxylon*), Achar (*Buchnannia lan- zan*), Mahua (*Madhuca indica*) are important sources of non-timber forest products. The fruits collected from these species and the firewood collected from the	Erratic rainfall patterns	Significant loss of agricultural crop production.	Development of a diverse livelihood portfolio for a poor rural household is resilience strategy.	While investing in local climate adaptation for rural communities, it is important to note that their vulnerability does not simply originate from weather shocks but also from fluctuations in government policies, which enables or

No.	Authors	Year	Study Title	Study Location	Target food system	Backbone ecosystem services (ES)	Natural hazards	Consequences to food systems	Resilience Interventions based on ES	Outcomes
						forest, constitute roughly 25% of their overall livelihood income.				constrains much of their livelihood capabilities.

No.	Authors	Year	Study Title	Study Location	Target food system	Backbone ecosystem services (ES)	Natural hazards	Consequences to food systems	Resilience Interventions based on ES	Outcomes
36	Parven A, Pal I, Hasan MS.(Parven, Pal, & Hasan, 2021)	2021	Ecosystem for disaster risk reduction in Bangladesh: A case study after the Cyclone "Aila".	Satkhira and Khulna districts in the southwestern coastal region of Bangladesh.	Small-scale farmers.	Livelihoods of people are totally dependent on ecosystem services of the Sundarbans forest.	Cyclone Aila (May 2009)	Crop and aquaculture production are highly affected. The saline water intrusion flooded the households, agriculture fields, ponds. Hundreds of livestock have been reported dead. Sundarbans-dependent people were unable to enter the	Forest-fish-fruit (FFF) model is an adaptation response that helps to reduce the risk of locals. The local people use the mangrove leaves as the source of the house roof and structure to build disaster-resilient settlement in coastal wetland areas. Floating seedbed and	FFF model is a long-term investment for income generation that is also a diversification of livelihoods. To receive multiple ecosystem services, it is necessary to improve ecological engineering systems. Besides

No.	Authors	Year	Study Title	Study Location	Target food system	Backbone ecosystem services (ES)	Natural hazards	Consequences to food systems	Resilience Interventions based on ES	Outcomes
								forest for at least 6 months for their livelihoods.	vegetable production. Salt-tolerant rice cultivation. Crab fattening for regular income in tidal inundation and waterlogged saline conditions.	this, it is important to make sure that ecosystem engineering provides a maximum amount of services and is consumed by the people at different labels and scales.

No.	Authors	Year	Study Title	Study Location	Target food system	Backbone ecosystem services (ES)	Natural hazards	Consequences to food systems	Resilience Interventions based on ES	Outcomes
37	Mutie FM, Rono PC, Kathambi V, Hu G-W, Wang Q-F. (Mutie, Rono, Kathambi, Hu, & Wang, 2020)	2020	Conservation of Wild Food Plants and Their Potential for Combatting Food Insecurity in Kenya as Exemplified by the Drylands of Kitui County.	Kitui county, dryland area of 30,496 km^2, Kenya.	Mixed farming which involves growing a variety of crops and keeping livestock. Cattle are kept as a security against famine. Kitui county has a high diversity of local foods in Kenya, including cultivated food crops.	Wild food plants: fruits, vegetables, nuts, cereals, roots, and tubers.	Climate change – frequent droughts.	Poor agricultural potential. Poor grazing resources and water quality make pastoralism increasingly difficult.	Livelihood diversification by means of wild plants is a strategy to cope with climatic uncertainties. A total of 199 wild plants have the potential of being utilized as food in different ways: fruits, vegetables, beverages, food additives, starch foods, seed foods, gums and	Prioritizing on proper harvesting, storage, and marketing of wild foods produced seasonally in large quantities might be an important step in maximizing the nutritional benefits of dryland communities.

No.	Authors	Year	Study Title	Study Location	Target food system	Backbone ecosystem services (ES)	Natural hazards	Consequences to food systems	Resilience Interventions based on ES	Outcomes
									resins,and others.	
