# Supplementary figures and images for: Considering Ecosystem Services in Food System Resilience
Source: Int J Environ Res Public Health. 2022 Mar 19;19(6):3652. doi: 10.3390/ijerph19063652 (PMC8954919; doi:10.3390/ijerph19063652)

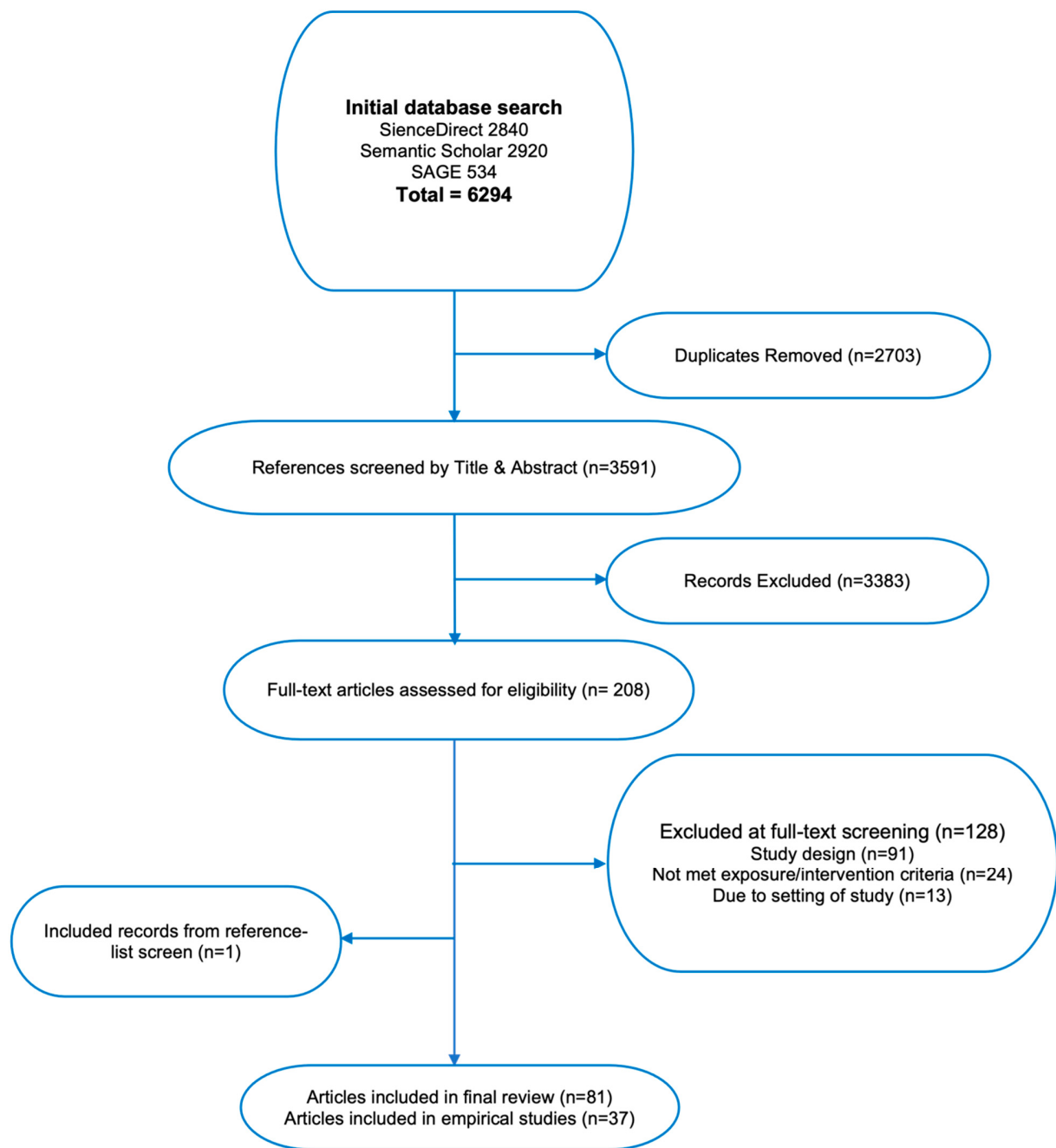

**Figure S1. PRISMA chart showing a study number at each stage of the review process**

Supplement: Supplementary file 1 [file ijerph-19-03652-s001.zip › Figure S1_PRISMA_chart.pdf]
